# The mTOR-Dop1a-Agpat2 axis regulates nuclear phospholipid homeostasis

**DOI:** 10.1016/j.isci.2026.115860

**Published:** 2026-04-22

**Authors:** Hirotaka Ariyama, Atsushi Tsukamura, Satoko Miyatake, Satoko Okado, Itsuki Itabashi, Ami Ogura, Atsunobu Suzuki, Hyuga Kurakawa, Yuki Sakaguchi, Yuhki Nakatake, Ryunosuke Sanada, Ichiro Terakado, Eriko Koshimizu, Takeshi Mizuguchi, Keisuke Hamada, Kazuhiro Ogata, Eiji Nakagawa, Takafumi Sakakibara, Manabu Shirai, Yoshitaka Fujihara, Mukhtar Ullah, Mathieu Quinodoz, Carlo Rivolta, Abdul Ghafoor Khan, Muhammad Nadeem Khan, Muhammad Ansar, Erica H. Gerkes, Tuula Rinne, Alexander P.A. Stegmann, Margje Sinnema, Malak Ali Alghamdi, Essa Alharby, Reham M. Balahmar, Naif A.M. Almontashiri, Sarah Baer, Amélie Piton, Carla Díes Curià, Sandra Mercier, Benjamin Cogné, Patrick Yap, Shin-ya Morita, Akiyoshi Kakita, Mitsuhiro Kato, Yoshihiro Maruo, Naomichi Matsumoto, Masaki Mori

**Affiliations:** 1 Department of Pediatric Physiology, Innovation & Research Support Center, Graduate School, International University of Health and Welfare, Tokyo, Japan; 2 Department of Pediatrics, Shiga University of Medical Science, Otsu, Japan; 3 Department of Human Genetics, Yokohama City University Graduate School of Medicine, Yokohama, Japan; 4 Department of Clinical Genetics, Yokohama City University Hospital, Yokohama, Japan; 5 Department of Neurogenetics, Molecular Neuroscience Research Center, Shiga University of Medical Science, Otsu, Japan; 6 Research Center for Animal Life Science (RCALS), Shiga University of Medical Science, Otsu, Japan; 7 Department of Biochemistry, Yokohama City University Graduate School of Medicine, Yokohama, Japan; 8 Department of Child Neurology, National Center Hospital, National Center of Neurology and Psychiatry, Tokyo, Japan; 9 Department of Pediatrics, Nara Medical University, Kashihara, Japan; 10 Omics Research Center, National Cerebral and Cardiovascular Center, Osaka, Japan; 11 Department of Advanced Medical Technologies, National Cerebral and Cardiovascular Center, Osaka, Japan; 12 Institute of Molecular and Clinical Ophthalmology Basel, Basel, Switzerland; 13 Department of Ophthalmology, University of Basel, Basel, Switzerland; 14 Department of Genetics and Genome Biology, University of Leicester, Leicester, UK; 15 Bannu Medical College, MTI Bannu, Bannu, Pakistan; 16 Department of Ophthalmology, University of Lausanne, Jules-Gonin Eye Hospital, Fondation Asile des aveugles, Lausanne, Switzerland; 17 Advanced Molecular Genetics and Genomics Disease Research and Treatment Centre, Dow University of Health Sciences, Karachi, Pakistan; 18 University of Groningen, University Medical Center Groningen, Department of Genetics, Nijmegen, the Netherlands; 19 Department of Human Genetics, Donders Institute for Brain, Cognition and Behavior, Radboud University Medical Center, Nijmegen, the Netherlands; 20 Department of Clinical Genetics, Maastricht University Medical Center, Maastricht, the Netherlands; 21 Medical Genetics Division, Pediatric Department, College of Medicine, King Saud University, Riyadh, Saudi Arabia; 22 Center for Genetics and Inherited Diseases, Taibah University, Almadinah Almunwarah, Saudi Arabia; 23 Faculty of Applied Medical Sciences, Taibah University, Almadinah Almunwarah, Saudi Arabia; 24 Institut de Génétique et de Biologie Moléculaire et Cellulaire, Illkirch, France; 25 Department of Pediatric Neurology, Strasbourg University Hospital, Strasbourg, France; 26 Laboratoire de Diagnostic Génétique, Institut de Génétique Médicale d'Alsace (IGMA), Hôspitaux Universitaire de Strasbourg, Strasbourg, France; 27 Institut de Génétique et de Biologie Moléculaire et Cellulaire, Illkirch, France; 28 IMPaCT-Genómica - Enfermedades Raras, Servei de Genètica, Hospital del Mar, Universitat Pompeu Fabra (UPF), Barcelona, Spain; 29 Service de génétique médicale, CHU de Nantes, Nantes, France; 30 Institut du thorax, INSERM, CNRS, Nantes Université, Nantes, France; 31 Genetic Health Service New Zealand (Northern Hub), Auckland City Hospital, University of Auckland, Auckland, New Zealand; 32 Faculty of Medicine and Health Sciences, University of Auckland, Auckland, New Zealand; 33 Department of Pharmacotherapeutics, Shiga University of Medical Science, Otsu, Japan; 34 Department of Pathology, Brain Research Institute, Niigata University, Niigata, Japan; 35 Department of Pediatrics, Showa Medical University, Tokyo, Japan; 36 Department of Rare Disease Genomics, Yokohama City University Hospital, Yokohama, Japan; 37 Advanced Pediatric Medicine, Tohoku University School of Medicine, Miyagi, Japan; 38 Department of Reproductive Medicine, Center for Regenerative Medicine, National Center for Child Health and Development (NCCHD), Tokyo, Japan; 39 Medical Genome Center, National Center of Neurology and Psychiatry, Kodaira, Japan; 40 Epilepsy Medical Center, Showa Medical University Hospital, Tokyo, Japan; 41 Center for Human Brain Resource Initiative (ChBRI), Niigata University, Niigata, Japan

**Keywords:** biological sciences

## Abstract

The molecular mechanisms regulating the phospholipid (PL) metabolism in the nucleus remain to be elucidated. Here, we describe the role of Dop1a in controlling PL abundance in nuclear membranes (NMs) under the control of mTOR signaling. A shortage of lysophosphatidic acid (LPA) triggers the rapid localization of Dop1a to the nuclear pore complexes (NPCs), where Dop1a suppresses PL synthesis by binding to AGPAT2 (1-acylglycerol-3-phosphate O-acyltransferase 2), which is also localized at the NPCs. Loss of *Dop1a* results in elevated PL production, which leads to the formation of nuclear lipid droplets (nLDs). The titration of PL abundance is coordinated with proper cell cycle entry by Dop1a that restricts nuclear accumulation of CDK2. Thus, Dop1a safeguards cell division via surveilling the PL supply. Dop1a is highly expressed in neurons and is essential for neurobehavioral development in mice. *DOP1A* mutations have been identified in patients with neurodevelopmental disorders (NDDs). Thus, the proper function of Dop1a is crucial for the proper development of the nervous system.

## Highlights

• LPA deprivation drives Dop1a to nuclear pores via Nup153 binding
• NPC-bound Dop1a suppresses Agpat2-driven nuclear phospholipid synthesis
• Dop1a limits CDK2 nuclear entry to couple lipid supply with cell cycle
• Human DOP1A mutations disrupt NPC targeting and drive neural disorders

## Graphical abstract

**Figure:**
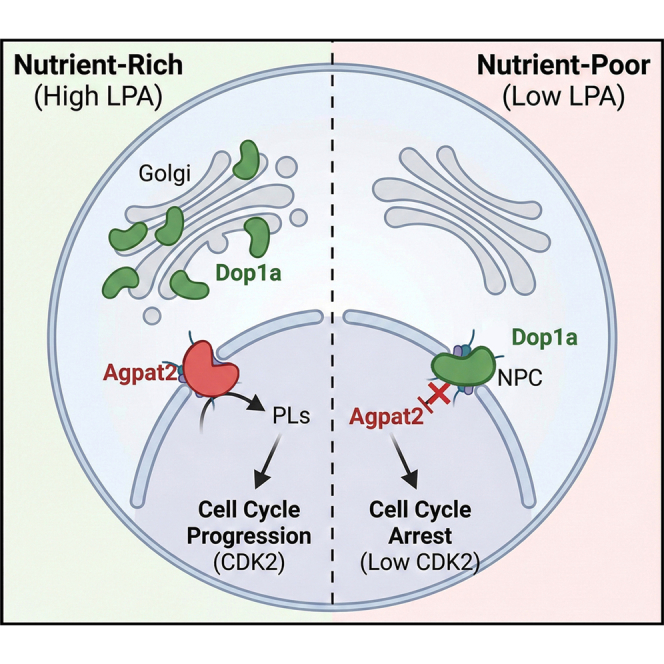

## Introduction

The nuclear membranes (NMs) are unique organelles of eukaryotic cells that protect the chromosomes and form a specialized milieu for genomic functions.^1^^,^^2^ The quantity of NM is tightly regulated along the cell cycle, involving its dispersal and reformation, warranting the proper functions of daughter nuclei.^3^^,^^4^ The precise machinery for sensing and regulating the NM quantity remains unclear.^5^ Failure of the phospholipid (PL) supply compromises the NM integrity, rendering the life system vulnerable. The regulatory mechanisms of how the PL is titrated in different nutritional availability remain elusive.

Biological membranes are synthesized by a group of enzymes engaged in PL synthesis.^6^^,^^7^^,^^8^ The NMs consist of an outer nuclear membrane (ONM) and an inner nuclear membrane (INM), and recent studies in *S. cerevisiae* revealed that lipid metabolism is active in the INM, which is crucial for the maintenance of lipid homeostasis in the nucleus and nuclear architecture.^9^^,^^10^^,^^11^ Whereas endoplasmic reticulum (ER) lipid metabolism is well-characterized, nuclear lipid synthesis is less understood. In the ER, PL synthesis is orchestrated by enzymes including those involved in the Kennedy pathway, which is responsible for the *de novo* synthesis of major PL species such as phosphatidylcholine (PC) and phosphatidylethanolamine (PE).^12^^,^^13^ A key enzyme in the upstream glycerophospholipid biosynthesis pathway, AGPAT2 (1-acylglycerol-3-phosphate O-acyltransferase 2), catalyzes the conversion of lysophosphatidic acid (LPA) to phosphatidic acid (PA), a central precursor for the synthesis of various PLs. A recent study discovered that lipid metabolic enzymes Ascl3, Gpat3, Gpat4, Dgat1, Dgat2, and Agpat2 exist in the INM and are engaged in the formation of nuclear lipid droplets (nLDs).^14^ However, a more comprehensive understanding of nuclear PL metabolism is needed to gain insight into the physiological relevance of the PL environment in the nucleus. The precise interplay between nuclear metabolism and cellular processes, such as cell division, has recently been described, indicating that the PL supply must be fine-tuned to extracellular environments.^15^ Indeed, the PL profile is dynamic and amenable to changes brought about by the rapidity of cell cycles and in cancerous tissues.^16^^,^^17^^,^^18^^,^^19^^,^^20^^,^^21^

Nuclear pore complexes (NPCs) are highly dynamic structures that disassemble and reassemble during the cell cycle.^22^^,^^23^^,^^24^^,^^25^ NPCs are formed at the attachment sites of the ONMs and INMs, functioning as gatekeepers that regulate the transport of molecules between the cytoplasm and nucleoplasm.^26^ Nup153 is a component of the basket structure that resides on the inner side of the NPC and plays a role in the selective incorporation of cytoplasmic molecules.^27^^,^^28^^,^^29^

The *DOP1A* gene (NC_000006.12) is well-conserved from budding yeast to humans,^30^ with an undetermined biological role beyond its role in vesicle transport via binding affinity to phosphatidylinositol 4-phosphate (PI4P) and interaction with Mon2 and the soluble N-ethylmaleimide-sensitive factor attachment protein receptor (SNARE) complex.^31^ Dop1a is a large, evolutionarily conserved protein (∼277 kDa), yet its physiological role remains poorly understood.

In this study, we illustrate the role of the LPA-mTOR-Dop1a-Agpat2 axis in maintaining nuclear PL homeostasis. LPA shortage triggers the NPC targeting of Dop1a that also interferes with the nuclear accumulation of CDK2, thereby ensuring safe entry into the cell cycle according to LPA availability. Insufficient expression of Dop1a causes maldevelopment of neuronal systems, thus illuminating the physiological role of nuclear PL regulation.

## Results

### Dop1a localizes to NPCs upon LPA shortage

**Figure 1:**
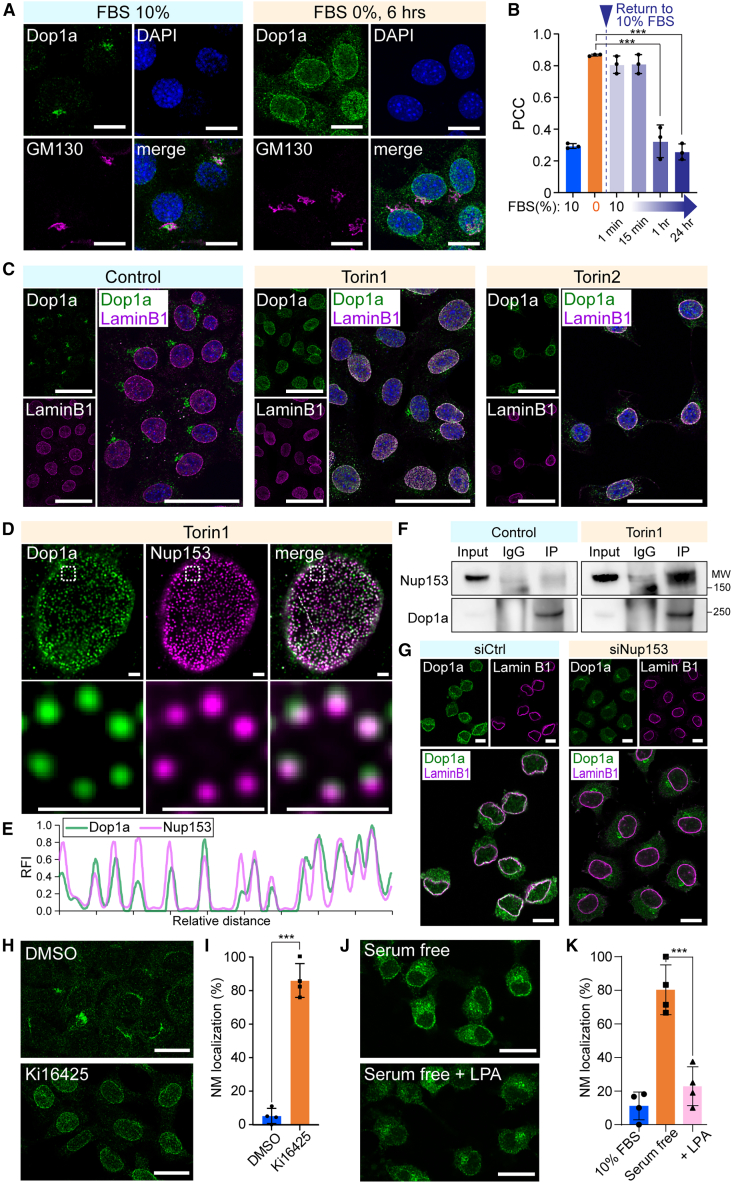
DOP1A localizes to NPC upon mTOR inhibition in a Nup153-dependent manner (A) Immunostaining of Dop1a in Neuro2a cells cultured with or without serum starvation for 6 h. The Golgi apparatus was labeled with an anti-GM130 antibody, and nuclei were visualized with DAPI. Scale bars, 10 μm. (B) Colocalization of Dop1a and Lamin B1 in Neuro2a cells subjected to serum starvation and re-feeding. Cells were cultured in 10% FBS, serum-starved for 24 h (0% FBS), and subsequently returned to 10% FBS for the indicated time points to assess the reversibility of Dop1a NM localization. Bars represent Pearson’s correlation coefficient (PCC) between Dop1a and Lamin B1 fluorescent signals. *n* = 3 biological replicates. (C) Dop1a localization in cells treated with Torin1 or Torin2. NMs were stained with the Lamin B1 antibody. Nuclei were stained with DAPI. Scale bars, 30 μm. (D) Super-resolution microscopy of Torin1-treated cells showing Dop1a localization at NM substructures. NPCs were stained with a Nup153 antibody, and nuclei were counterstained with DAPI. The dashed line indicates the position used for the linescan shown in (E). Scale bars, 1 μm. (E) Colocalization analysis of Dop1a and Nup153. Fluorescence intensity along the dashed line in (D) was quantified and plotted as relative values. (F) Co-immunoprecipitation (IP) analysis performed with the Dop1a antibody. Lysates of cells treated or not with Torin1 were incubated with the Dop1a or control-IgG antibody to collect proteins interacting with Dop1a. MW, molecular weight (kDa). (G) Localization of Dop1a in Neuro2a cells transfected with control or Nup153 siRNA. The NM was shown by Lamin B1 immunostaining. Scale bars, 10 μm. (H) Subcellular localization of Dop1a in cells exposed to treatment with Ki16425 or DMSO. Scale bars, 20 μm. (I) Frequency of cells exhibiting NM localization following Ki16425 or DMSO treatment (*n* = 3 biological replicates; 71–92 cells analyzed per replicate). (J) Dop1a localization under LPA supplementation. Scale bars, 20 μm. (K) Frequency of cells showing NM localization with or without LPA supplementation. (*n* = 3 biological replicates; 48–92 cells were analyzed per replicate.) Data are represented as means ± SEM. ∗∗∗*p* < 0.001, unpaired two-tailed Student’s *t* test.

Upon cellular imaging analysis, we found that Dop1a changed its localization from the Golgi apparatus to the NMs upon serum starvation (Figure 1A). The NM localization of Dop1a was reversed within 1 h when cells were refed (Figure 1B). Treatment with Torin1, Torin2, and rapamycin, inhibitors of the mTOR signaling pathway, recapitulated the NM localization, suggesting a role for the mTOR pathway as an upstream determinant of Dop1a localization (Figures 1C and S1A). The Dop1a protein levels were not affected by serum starvation (Figure S1B) or Torin1 treatment (Figure S1C). The specificity of the immunostaining was confirmed in *Dop1a* knockout (KO) mouse embryonic fibroblasts (MEFs, Figures S1D and S1E). Dop1b, a homolog of Dop1a, did not show NM localization, indicating that our observation was specific to Dop1a (Figure S1F). We next sought to determine which substructures of the NM Dop1a associates with. To this end, we examined whether Dop1a localizes to nuclear pore complexes (NPCs) using an antibody against Nup153, a component of the nuclear pore basket that yields clear signals in immunofluorescence analyses. Super-resolution structured illumination microscopy (SIM)^32^ revealed that Dop1a signal overlapped with that of Nup153, a constitutive component of the NPC basket structure, selectively in the Torin1-treated condition (Figures 1D and 1E), and not in the DMSO-control condition (Figure S1G), suggesting that Dop1a localizes to NPCs. Co-immunoprecipitation assay revealed that Dop1a and Nup153 interacted selectively upon Torin1 treatment (Figure 1F). Upon *Nup153* depletion (Figure S1H), Dop1a failed to localize to the NM, demonstrating that Dop1a NM localization is Nup153-dependent (Figure 1G). By contrast, Dop1a was dispensable for proper Nup153 localization at the NM (Figure S1I). We also assessed the types of mTOR-sensitizing nutrients with which Dop1a function correlates. Among the compounds we tested for competency to induce NM localization, Ki16425, an inhibitor of LPA receptor (LPAR) function, provoked the NM localization of Dop1a (Figures 1H and 1I). This finding suggests that the Dop1a NM location anti-correlates with the abundance of LPA contained in the serum. Indeed, the replenishment of LPA in the serum-starved condition significantly blocked the NM localization, corroborating that Dop1a relocalization is provoked by the shortage of LPA abundance (Figures 1J and 1K). Together, Dop1a exhibits reversible localization to the NPCs upon LPA shortage.

### *Dop1a* depletion enhances PL synthesis

**Figure 2:**
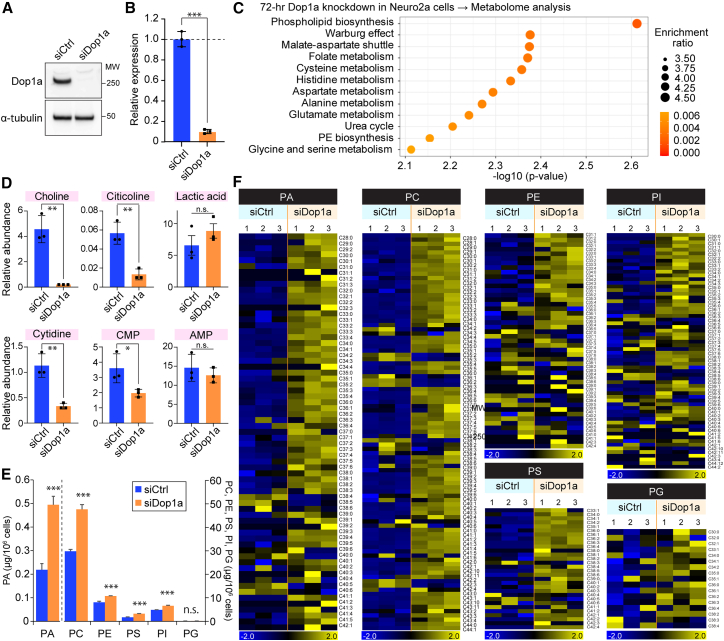
DOP1A lowers PL abundance (A) Western blot analysis of Dop1a to assess knockdown efficiency in Neuro2a cells. α-Tubulin was used as a loading control. MW, molecular weight (kDa). (B) The knockdown efficiency for *Dop1a*. Gene expression levels were normalized to *Tubb5* (*n* = 3). (C) Metabolome analysis of serum-starved Neuro2a cells depleted of *Dop1a* for 72 h. Metabolite set enrichment analysis (MSEA) was used to identify over-represented metabolic pathways altered by *Dop1a* knockdown. Dot size shows the enrichment ratio (observed/expected hits), and dot color reflects pathway significance (*p* value). The *x* axis indicates –log_10_(*p* value). PE, phosphatidylethanolamine. (D) The abundance of metabolites associated with PL metabolism measured by LC-MS. The data were normalized to the number of cells. The levels of lactate and AMP, which are not substrates for PL metabolism, were shown for comparison (*n* = 3). CTP, cytidine monophosphate. AMP, adenosine monophosphate. (E) PL global analysis in cells depleted of *Dop1a* or left non-depleted, followed by Torin1 treatment. After siRNA transfection for 16 h, cells were treated with Torin1 or left untreated for 48 h. The data were normalized to the number of cells (*n* = 3). PA, phosphatidic acid; PC, phosphatidylcholine; PS, phosphatidylserine; PI, phosphatidylinositol; PG, phosphatidylglycerol. (F) Heatmap of PL molecular species in siCtrl and siDop1a cells (*n* = 3 biological replicates each). Values are shown as *Z* scores (standard scores) calculated for each lipid species across all samples. Data are represented as means ± SEM. ∗*p* < 0.05, ∗∗*p* < 0.01, and ∗∗∗*p* < 0.001, unpaired two-tailed Student’s *t* test.

Based upon the finding that Dop1a functions downstream of the LPA-mTOR axis, we sought to identify the functional target of Dop1a. We examined the effects of Dop1a loss in metabolism (Figures 2A and 2B), postulating that Dop1a is responsible for a specific branch of mTOR-regulated cell metabolism. The comprehensive metabolome analysis via liquid chromatography-mass spectrometry (LC-MS) under serum-starved conditions revealed that, in *Dop1a*-depleted cells, “PL biosynthesis” was the metabolic pathway that was most significantly affected (Figure 2C). In contrast, under normal nutritional conditions, *Dop1a* depletion had no obvious effect on the metabolome, suggesting the PL regulatory function of Dop1a is dependent on its NPC localization (Figure S2A). Comparison of individual metabolites showed that precursors for PL biosynthesis, such as choline, citicoline, cytidine, and cytidine monophosphate (CMP), fell short as a result of *Dop1a* depletion (Figure 2D), suggesting that *Dop1a* depletion causes the exhaustion of PL precursor metabolites. We next tested how *Dop1a* depletion affects the abundance of PLs via LC-MS. *Dop1a* depletion resulted in the overproduction of PLs in cells, with significant increases in PA, PC, PE, phosphatidylserine (PS), and phosphatidylinositol (PI) (Figure 2E). In contrast, this PL overproducing effect was not observed in DMSO-treated conditions, indicating NM localization is essential for regulating PL abundance (Figures S2B and S2C). The influence of *Dop1a* depletion was observed in a broad range of PL species, revealing the role of Dop1a in regulating the abundance of PLs in response to nutritional availability (Figure 2F). Together, these findings suggest that the primary function of Dop1a is to regulate PL abundances negatively in response to nutrient deprivation.

### Dop1a targets Agpat2 to regulate nuclear PLs

**Figure 3:**
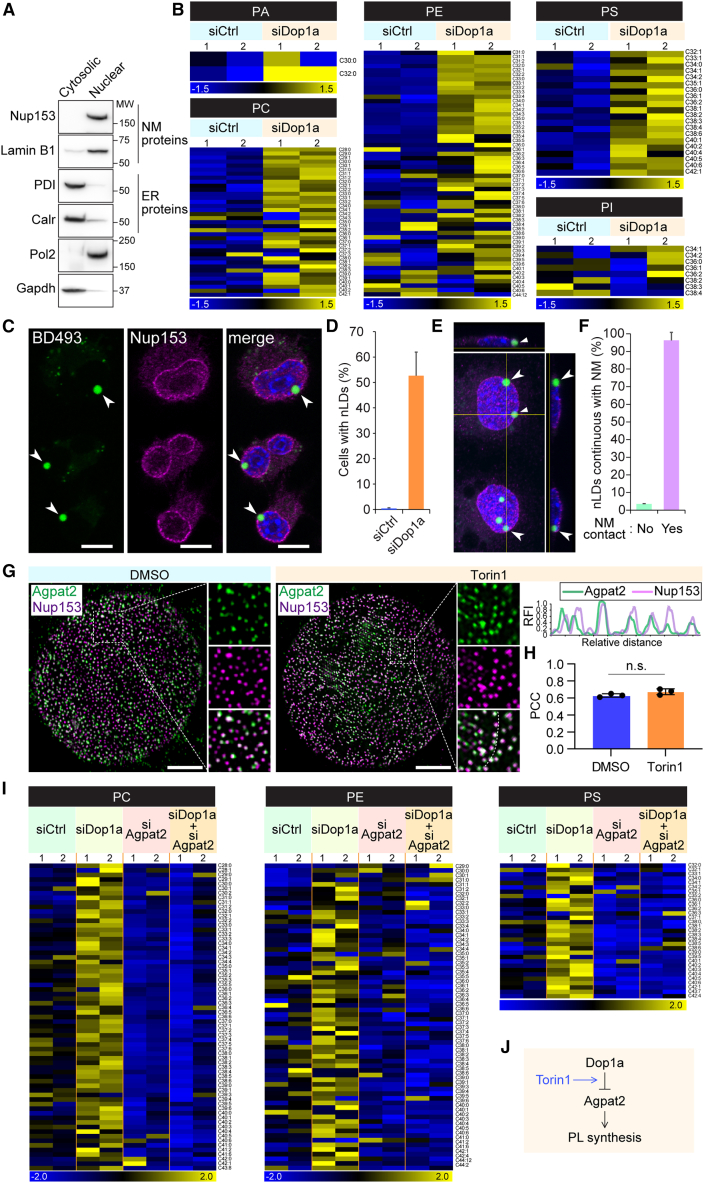
DOP1A targets Agpat2 to regulate PL synthesis (A) Western blot of the cytoplasmic fraction enriched in ER components and the nuclear fraction containing NM components in Neuro2a cells. (B) PL profiles of the nuclear fraction. The nuclei of control and *Dop1a* knockdown cells cultured in the presence of Torin1 were subjected to LC-MS. Values are shown as *Z* scores calculated for each lipid species across all samples. (C) Visualization of nuclear lipid droplets (nLDs) in the *Dop1a*-depleted cells treated with Torin1. The nLDs were stained with Bodipy 493/503 (BD493). Scale bars, 10 μm. (D) Frequency of cells exhibiting nLDs (*n* = 267 and 55 cells). (E) The position of nLD within the nucleus observed by the *z* axis stacking. (F) The frequency of nLDs is showing continuity with the NM (*n* = 101 and 99 cells). (G) Super-resolution microscopy of Agpat2 and Nup153. The linescan was performed along the dashed line indicated in the inset image. Scale bars, 2 μm. (H) Coloc2 analysis of Agpat2 and Nup153 fluorescent signals in the cells treated with Torin1 or DMSO. PCC, Pearson’s correlation coefficient; n.s., not significant. (I) The PL global analysis performed in Neuro2a cells after depletion of both Dop1a and Agpat2, followed by Torin1 treatment. Values are shown as *Z* scores calculated for each lipid species across all samples. (J) Schematic for the coregulatory module of Dop1a and Agpat2 over the PLs. Data are represented as means ± SEM. ∗*p* < 0.05, ∗∗*p* < 0.01, and ∗∗∗*p* < 0.001, unpaired two-tailed Student’s *t* test.

Based on our observation that Dop1a reduces PL abundance upon NM localization, we next examined the impact of Dop1a loss on nuclear lipid homeostasis. Cytosolic and nuclear fractions were prepared from Neuro2a cells, and the efficiency of fractionation was confirmed by immunoblotting (Figure 3A). Quantitative PA and PC assays using these fractionated samples revealed that *Dop1a* depletion significantly increased PA and PC levels in the nuclear fraction, particularly under Torin1 treatment (Figures S3A and S3B). Consistent with these findings, PL MS analysis further demonstrated that *Dop1a* knockdown resulted in a broad increase in multiple PL species, including PA, PC, PE, and PS, within the NM-containing fraction (Figure 3B). Together, these results indicate that Dop1a limits PL at the NM and functions as a regulator of nuclear PL homeostasis. Surplus PLs are converted to neutral lipids. Indeed, staining with neutral lipid probe Bodipy 493/503 revealed the formation of nLDs in *Dop1a*-depleted cells (Figures 3C and 3D). The nLDs appeared to harbor a continuum to the NMs, suggesting that the neutral lipids were synthesized from the NMs (Figures 3E and 3F).

We further analyzed how Dop1a affects the PL abundance in the nucleus. Among the enzymes catalyzing PL metabolism, we found that Agpat2 showed localization to the NMs (Figure S3C). Agpat2 also showed colocalization to Nup153, as assessed via super-resolution microscopy (Figures 3G and 3H). Furthermore, the coimmunoprecipitation assay revealed that Dop1a physically interacted with Agpat2 selectively upon Torin1 treatment, suggesting that Agpat2 is the metabolic target of Dop1a (Figure S3D).

To examine whether Dop1a regulates nuclear PLs via Agpat2, we tested the influence of concomitant depletion of *Dop1a* and *Agpat2* on the PL abundance in the nucleus (Figure S3E). Upon *Dop1a* depletion, the abundance of PL species was enhanced, but with the concomitant depletion of *Agpat2*, the enhancing effects were wholly canceled (Figure 3I), establishing that Dop1a regulates nuclear PL abundance through Agpat2 (Figure 3J).

To further address whether the Dop1a-dependent regulation of PL synthesis reflects indirect effects of mTOR inhibition, we next examined the involvement of translational control and autophagy. Inhibition of cap-dependent translation with the eIF4E-eIF4G interaction inhibitor 4EGI-1 did not attenuate the increase in PL abundance induced by *Dop1a* knockdown (Figure S3F). Similarly, pharmacological blockade of autophagy using bafilomycin A1 (BafA1) failed to suppress the *Dop1a*-knockdown-associated PL elevation (Figure S3G). These results indicate that the effect of Dop1a on PL synthesis is not mediated by translational repression or autophagy downstream of mTOR, supporting a model in which Dop1a regulates PL abundance through an Agpat2-dependent mechanism.

### Dop1a stalls cell growth in response to mTOR activity

**Figure 4:**
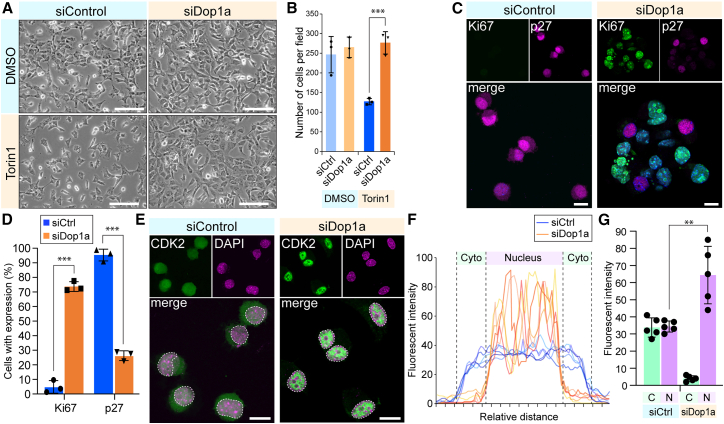
Dop1a depletion induces CDK2 nuclear localization and promotes cell growth (A) Phase-contrast images of NIH3T3cells depleted of Dop1a and treated with Torin1. Images were obtained 72 h after transfection. Scale bars, 100 μm. (B) The number of cells grown after the transfection of siRNAs and treatment with Torin1 (*n* = 3 biological replicates). (C) The immunostaining of Ki67 and P27 in NIH3T3 cells transfected with the siRNAs and treated with Torin1. Nuclei were stained with DAPI. Scale bars, 10 μm. (D) The frequency of cells with positive staining with Ki67 and P27 (*n* = 3 biological replicates). (E) The immunostaining of CDK2 in the NIH3T3cells transfected with the siRNAs and treated with Torin1. Dashed lines indicate the NMs. Scale bars, 10 μm. (F) Fluorescent intensity across the cells after the siRNA transfection and Torin1 treatment. Dop1a depletion induces the exclusive nuclear location of CDK2. (G) Quantification of cytoplasmic (C) and nuclear (N) fluorescent intensities in siCtrl- and siDop1a-transfected NIH3T3cells. Intensities were obtained from line-scan profiles across individual cells and averaged for the cytoplasmic and nuclear regions. Data are represented as means ± SEM. ∗∗*p* < 0.01, ∗∗∗*p* < 0.001, unpaired two-tailed Student’s *t* test.

Based on the findings that Dop1a regulates nuclear PL via the mTOR-LPA-Dop1a-Agpat2 axis, we next examined the cellular effects of *Dop1a* depletion. *Dop1a* depletion canceled the growth-suppressive effect of Torin1 in NIH3T3 cells (Figures 4A and 4B) and Neuro2a cells (Figures S4A and S4B), suggesting that Dop1a decelerates cell growth when mTOR signaling is inactivated. Indeed, immunostaining analysis revealed that *Dop1a* depletion induced the expression of Ki67 protein, indicating that Dop1a depletion promotes rigorous cell proliferation even in the presence of Torin1 (Figures 4C and 4D). Moreover, *Dop1a* depletion lowered the number of cells expressing p27^Kip1^, a CDK inhibitor, corroborating that Dop1a loss enhances cell growth property (Figures 4C and 4D). Further analysis of the upstream growth regulator uncovered that the nuclear accumulation of CDK2, a license protein of G1-S transition, was enhanced by *Dop1a* depletion (Figure 4E). Quantitative analysis of CDK2 fluorescence in cytoplasmic and nuclear compartments revealed a significant increase in nuclear CDK2 levels upon Dop1a depletion in NIH3T3 cells (Figures 4F and 4G). These results show that *Dop1a* depletion causes nuclear localization of CDK2 and enhanced cell proliferation.

### *Dop1a* deficiency causes PL overabundance and altered cell composition in the brain

**Figure 5:**
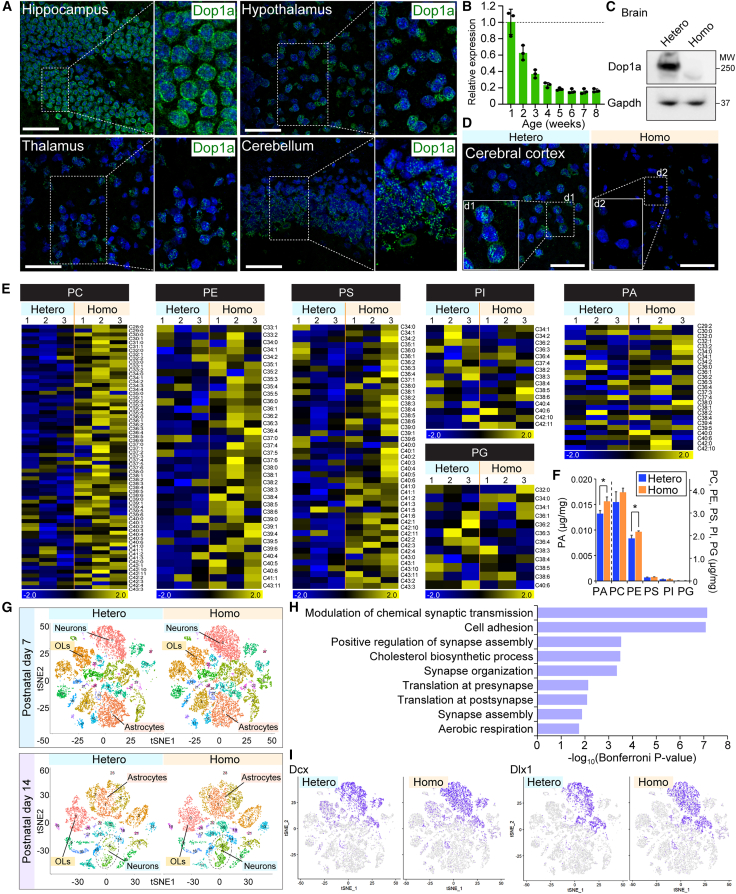
*Dop1a* deficiency causes neuronal overgrowth in mice (A) Immunostaining of Dop1a in the brain tissues from mice on P7. Tissue sections were made to analyze the expression patterns of Dop1a. Scale bars, 100 μm. (B) Age-dependent expression of *Dop1a* in the mouse cerebral cortex. Data were normalized to *Gapdh* (*n* = 3). (C) Generation of *Dop1a* KO mice. Dop1a protein expression in the cerebral cortex of *Dop1a* heterozygous and homozygous KO mice. GAPDH was used as the loading control. (D) Immunofluorescence of Dop1a in the cerebral cortex of *Dop1a* heterozygous and homozygous KO mice. Nuclei were stained with DAPI. Scale bars, 100 μm. (E) Heatmap showing the PL abundance in the cerebral cortex. Values are shown as *Z* scores calculated for each lipid species across all samples. (F) PL-MS performed with cerebral cortices of *Dop1a* heterozygous and homozygous KO mice at P14 (*n* = 3). (G) The single-cell RNA-seq analysis showing the *t*-SNE clustering results obtained from heterozygous and homozygous KO mice on P7 (top) and P14 (bottom). The clusters corresponding to neurons, astrocytes, and oligodendrocytes (OLs) are indicated. (H) Gene ontology enrichment of differentially expressed genes between heterozygous and homozygous cerebral cortex. The top enriched biological process categories are shown. (I) Feature plot visualizations of *Dcx* and *Dlx1* expression in P7 cortical cells. Both markers show predominant enrichment in the same neuronal cluster that was used for quantitative analysis. Data are represented as means ± SEM. ∗*p* < 0.05, ∗∗*p* < 0.01, and ∗∗∗*p* < 0.001, unpaired two-tailed Student’s *t* test.

We next asked about the biological relevance of Dop1a function in mice. Dop1a protein is abundantly expressed in the hippocampus, hypothalamus, thalamus, and cerebellum (Figure 5A), and the expression levels were higher on postnatal days 1 (P1) and 7 (P7), compared to day 56 (P56), implying its role in neural development (Figure 5B). Neonatal cortical neurons showed the NM localization of Dop1a when treated with Torin1, reveals that Dop1a is capable of NM localization responding to mTOR inactivation in the neurons (Figure S5A). We established *Dop1a* KO mice (Figures 5C, S5B, and S5C), which showed no appreciable signal in the cerebral cortex, where Dop1a signals were observed in the heterozygous counterparts (Figure 5D). We next examined whether heterozygous loss of *Dop1a* affects cellular physiology. Growth analysis of MEFs derived from wild-type (WT), heterozygous, and homozygous *Dop1a* KO mice showed that, while homozygous *Dop1a* deficiency increased cell number, heterozygous MEFs grew comparably to WT controls (Figure S5D). These results indicate that partial loss of *Dop1a* does not measurably affect basal cell growth, supporting the use of heterozygous mice as controls for comparison with homozygous *Dop1a* KO mice. The PL global analysis of cortical brain tissues from P14 mice revealed that the amount of PLs, such as PA and PE, was significantly increased in Dop1a homozygous KO mice compared to heterozygous KO mice, consistent with the *in vitro* findings (Figures 5E and 5F). These findings imply that PL synthesis is overdriven in the cerebral cortex of *Dop1a* KO mice.

To evaluate whether Dop1a deficiency affects cellular states *in vivo*, we performed single-cell RNA sequencing (scRNA-seq) analysis of cerebral cortices from *Dop1a* KO and heterozygous control mice at P7 and P14. Dimensional reduction and cell-type annotation revealed that neuronal clusters occupied a larger proportion in *Dop1a* KO cortices compared with heterozygous controls, a trend consistently observed at both developmental stages (Figure 5G). Quantitative analysis confirmed an increased proportion of neurons accompanied by reduced proportions of astrocytes and oligodendrocytes (Figure S5E). Gene ontology analysis of differentially expressed genes identified enrichment of pathways related to the modulation of chemical synaptic transmission (Figure 5H), prompting us to examine neuronal populations further. Feature plots of neural lineage-associated markers, including *Dcx* and *Dlx1*, revealed a modest expansion of neural progenitor-like cell populations in *Dop1a* KO cortices (Figure 5I). Heatmap visualization of significantly altered genes demonstrated broad transcriptional changes in neuronal clusters upon *Dop1a* loss (Figure S5F). Consistently, gene signature analyses indicated a shift toward S and G2/M phase cell-cycle programs in *Dop1a*-deficient neurons (Figure S5G), whereas apoptosis-related gene signatures were unchanged (Figure S5H). Together, these analyses indicate that *Dop1a* deficiency alters neuronal composition and transcriptional programs in the cerebral cortex, accompanied by enhanced cell-cycle activity.

### *DOP1A* variants identified in patients with NDDs

**Figure 6:**
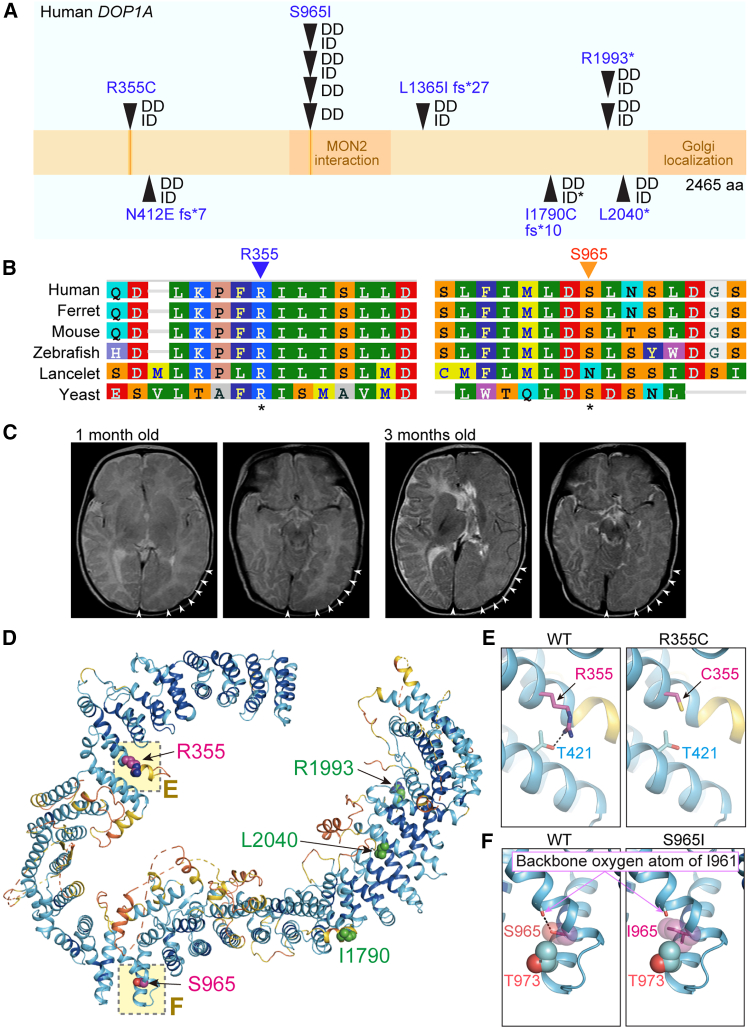
DOP1A mutation causes a neuronal developmental disorder (A) Location and interspecies conservation of amino acids mutated in patients. DD, developmental delay; ID, intellectual disability. (B) Evolutionary conservation of amino acids at around R355 and S965. (C) The magnetic resonance imaging (MRI) images of the patient harboring the DOP1A-R355C mutation taken at 1 and 3 months after birth. Arrowheads indicate the region affected by hemimegalencephaly. (D) An AlphaFold2 model of the DOP1A structure. The residues with missense variations, R355 and S965, are located in regions of high confidence in the calculation (magenta spheres). The residues I1790 (frameshift), R1993, and L2040 (nonsense) are green spheres. The regions indicated as dashed lines are disordered. (E) Close-up view of the region around R355. A hydrogen bond involved in the R355 of the WT (left) is missing in the R355C variant (right). The side chains of the residues involved in the hydrogen bond are displayed as sticks. (F) Close-up view of the region around S965. In the wild-type, S965 forms a hydrogen bond with the backbone carbonyl oxygen of I961 and makes van der Waals contact with T973 (left). The S965I variant (right) shows a loss of hydrogen bonding and van der Waals contact.

We next sought to analyze the effect of *DOP1A* variation in humans. By the worldwide collaborative study through GeneMatcher,^33^ rare *DOP1A* (NM_015018.4; Online Mendelian Inheritance in Man [OMIM] 616823) variants were identified in eleven patients from eight families with neurodevelopmental disorders (NDDs) presenting intellectual disability (ID) and developmental delay (DD, Figures 6A, S6A; Table S1. See Supplemental Information for detailed clinical summary). Five patients had each different *de novo* heterozygous variants (R355C, N412Efs∗7, L1365Ifs∗27, I1790Cfs∗10, and L2040∗), and six patients from two families carried homozygous variants (S965I and R1993∗). Of the seven variants identified, five were nonsense or frameshift mutations, and two were missense mutations (R355C and S965I). All variants are absent in the publicly available genome variation database, except for L1365Ifs∗27, which is found only rarely in 1 individual of African descent (1/152068 alleles, 0.0006576%). The gnomAD analysis indicated that DOP1A is intolerant to both missense variants (Z score = 3.32) and predicted loss-of-function (pLoF) variants, with a pLoF observed/expected (o/e) ratio of 0.25 and a probability of loss-of-function intolerance (pLI) of 1, implying that DOP1A variants underlie the abnormal phenotype. Together, all the identified variants were considered pathogenic or likely pathogenic based on the American College of Medical Genetics and Genomics/Association for Molecular Pathology (ACMG/AMP) guidelines (Table S2,.^34^ These patient findings corroborated the functional relevance of *Dop1a* in the development of the nervous system.

To examine the role of DOP1A in human neural lineage cells, we differentiated human induced pluripotent stem cells (iPSCs) into neural stem cells (NSCs) and performed RNAi-mediated depletion of *DOP1A*. *DOP1A* expression was efficiently reduced by two independent small interfering RNAs (siRNAs) (Figure S6B). Under mTOR inhibition with Torin1, *DOP1A*-depleted NSCs displayed increased cell numbers compared with control cells, as assessed by imaging-based cell counting (Figures S6C and S6D). These results indicate that *DOP1A* contributes to restraining NSC expansion under mTOR-inhibited conditions in human cells, consistent with the growth phenotypes observed in mouse models.

The findings that conserved amino acids were substituted in mutant *DOP1A* prompted us to investigate the structural relevance of the identified mutations in *DOP1A*. The two missense variations occurred in the highly conserved amino acids, R355 and S965 (Figure 6B). The R355C mutation caused hemimegalencephaly, a unilateral hyperplasia of the brain tissue, recapitulating the findings with the *Dop1a* mutation in mice (Figure 6C). AlphaFold2 modeling revealed R355 and S965 located in α-helixes (Figure 6D). R355 forms a hydrogen bond with T421 of the adjacent α-helix (Figure 6E), thus suggesting the mutation might destabilize the amino acid interaction. Also, S965 forms a hydrogen bond with the oxygen atom of the I961 backbone and makes van der Waals contact with T973 (Figure 6F). Thus, the S965I variation might destabilize the DOP1A structure by compromising the bonds with T973. Together, *DOP1A* variations found in NDD patients were considered to have a harmful effect on the physiological functions of DOP1A.

### The Dop1a NPC targeting underlies the behavioral development

**Figure 7:**
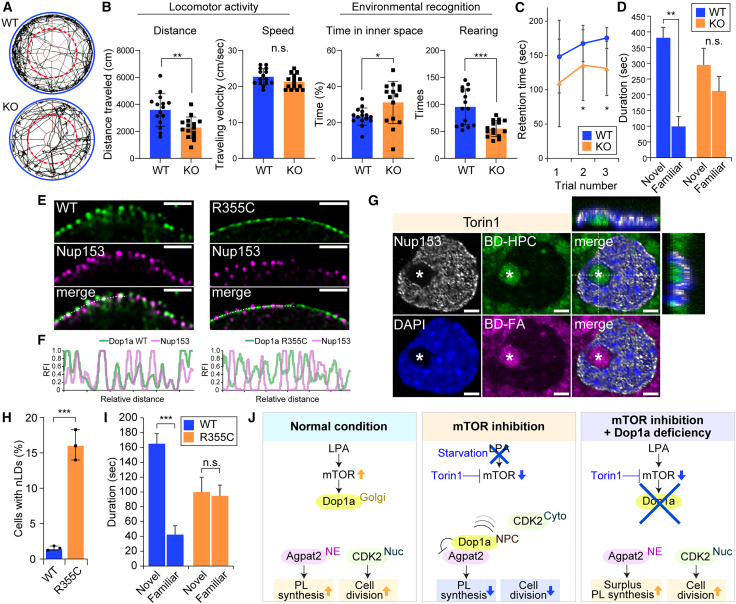
The R355C mutation causes aberrant localization of Dop1a and underlies abnormal behaviors (A) Representative trajectories of mice observed in the open field test. The red dashed line indicates the central area. (B) Quantitative analysis of the open field test with WT and *Dop1a* KO mice (*n* = 13, 14). (C) Wire-hang test to examine the muscle coordination of WT and *Dop1a* KO mice (*n* = 16, 19). The statistical significance was examined using a chi-squared test. ∗*p* < 0.05, and ∗∗∗*p* < 0.001. (D) Three-chamber sociality test with WT and *Dop1a* KO mice (*n* = 15, 15). (E) Subcellular localization of Dop1a-R355C protein. MEFs were isolated from the Dop1a-R355C mutant mouse fetuses and utilized to assess the localization of Dop1a-R355C. The line scan was performed along the dashed line indicated in the image. Scale bars, 1 μm. (F) The co-localization of Dop1a-R355C and Nup153 was analyzed as the co-occurrence of fluorescence signals. RFI, relative fluorescence intensity. (G) nLD formation in the MEFs harboring the Dop1a-R355C mutation. The MEFs were cultured in the presence of Torin1. Lipids were stained with Bodipy 493/503 and Bodipy fatty acid. The NM was stained with the Nup153 antibody. Scale bars, 2 μm. (H) Frequency of nLD-forming cells. (I) Three-chamber sociality test with WT and Dop1a-R355C mice (*n* = 10, 10). Unless indicated, data are represented as means ± SEM. ∗*p* < 0.05, ∗∗*p* < 0.01, and ∗∗∗*p* < 0.001, unpaired two-tailed Student’s *t* test. (J) Schematics describing the role of Dop1a in sensing LPA and in the coordination of PL synthesis and cell cycle entry.

The clinical findings of the patients with *DOP1A* variations prompted us to examine the neurodevelopmental phenotypes of the *Dop1a* mutant mice. We performed a battery of behavioral tests with the *Dop1a* mutant mice. In the open field test, the *Dop1a* KO mice showed decreased total migration, more frequent internal crossing (Figures 7A and 7B), and reduced rearing behavior (Figure 7B), suggesting impaired environmental recognition. The wire-hanging test showed poorer performance by KO mice than WT mice, indicating that *Dop1a* deficiency causes impaired motor coordination (Figure 7C). In the 3-chamber social interaction assay, WT mice exhibited the expected preference for a novel mouse, whereas *Dop1a* KO mice spent significantly longer time with the familiar mouse (Figure 7D). This abnormal preference indicates a decline in social recognition in *Dop1a* KO mice.

Finally, to examine the functional relevance of the amino acid substitutions identified in the patients, we established the mouse strains harboring the R355C variant (Figures S7A and S7B). The primary neurons isolated from the cortical tissues of WT and R355C mice were subjected to super-resolution microscopy, revealing that the Dop1-R355C protein failed to localize to the NPCs, though maintaining the association with the NMs (Figures 7E and 7F). This finding suggests that the Dop1-R355C protein interacts with the NMs but fails to localize selectively to the NPCs. The MEFs isolated from the Dop1a-R355C mice developed nLDs, which were hardly observed in the WT MEFs (Figures 7G and 7H). We analyzed the effects of the R355C mutation on the mouse behavioral development via the 3-chamber test. We observed impaired discrimination of novel and familiar mice, compared to WT, indicating the compromised behavioral development in the Dop1a-R355C mutant mice (Figure 7I).

## Discussion

Our study identifies Dop1a as an mTOR signaling-responsive regulator that relocates to NPCs to curb Agpat2-driven PL synthesis. We show that Dop1a localizes to NPCs via binding to Nup153 upon LPA deprivation and suppresses PL synthesis through direct interaction with Agpat2. *Dop1a* depletion leads to derepressed PL production, resulting in the formation of nLDs. NPC targeting of Dop1a inhibits CDK2 nuclear translocation, thereby coordinating PL synthesis with cell-cycle progression. Loss of *Dop1a* provokes neural cell overgrowth and compromises proper neural development in mice (Figure 7J).

Under normal conditions, Dop1a localizes to the Golgi apparatus, as previously reported,^31^ and changes its subcellular localization upon LPA deprivation. LPA binding to LPARs activates mTOR signaling,^35^^,^^36^ and inhibition of LPARs by Ki16425 facilitates NPC localization of Dop1a. These findings suggest that Dop1a functions under the control of LPA-mTOR signaling.

In our model, Dop1a limits PL abundance by suppressing Agpat2 activity. The slight reduction in *Agpat2* expression observed after *Dop1a* knockdown would not be expected to increase PL levels and therefore does not readily account for our findings. Instead, the data raise the possibility that activity-related aspects of Agpat2, rather than its transcriptional level, are influenced by Dop1a.

NMs are functional sites for lipid synthesis and play essential roles in maintaining the nuclear microenvironment.^9^^,^^14^ Our study reveals a previously unrecognized role of NPCs in PL metabolism and clarifies an indispensable role for the PL synthetic enzyme Agpat2 in regulating nuclear PL species. NPCs are multi-protein assemblies that undergo dynamic structural changes during the cell cycle, and our findings indicate that they function not only as transport conduits but also as active platforms for regulating lipid metabolism.

We further show that the R355C mutation disrupts the ability of Dop1a to localize to NPCs. NPC localization is essential for the PL-regulatory function of Dop1a, as the R355C mutation led to the accumulation of nLDs. This response is considered protective, as it converts excess PL-derived diacylglycerol into neutral lipids, thereby preventing diacylglycerol-induced lipotoxicity.^37^ These observations illustrate a function of Dop1a in maintaining an appropriate nuclear lipid environment through its action at NPCs.

The failure of Dop1a to relocalize to the NMs after Nup153 depletion is consistent with the established role of Nup153 in maintaining NPC integrity. Previous studies have shown that loss of Nup153 disrupts the nuclear basket, alters NPC distribution, and reduces nuclear import competence, indicating that NPCs are structurally compromised under these conditions.^27^ More recent work further demonstrates that Nup153 depletion perturbs nuclear envelope organization during telophase, leading to the mistargeting of multiple NPC-associated membrane proteins.^38^ Within this framework, the absence of Dop1a at NPCs supports the idea that Dop1a requires fully assembled and structurally intact NPCs for its recruitment.

NPC targeting of Dop1a prevents CDK2 from localizing to the nucleoplasm, thereby inhibiting progression from G1 to S phase. Notably, *Dop1a* depletion restores not only PL abundance but also cell proliferation under mTOR-inhibited conditions. This observation suggests that Dop1a functions upstream of PL metabolism and also upstream of pathways governing cell-cycle progression. Although the mechanisms linking PL synthesis to cell-cycle control remain unclear, these processes are likely to be functionally connected. One plausible link is the requirement for sufficient PL and membrane production to support organelle biogenesis during cell division. Because all membrane-bound organelles must be duplicated during proliferation, cells must secure an adequate supply of PLs. Under nutrient-limited conditions, Dop1a may suppress PL synthesis by targeting Agpat2 at the NM and may also contribute to quantitative regulation of membrane availability in relation to proliferative capacity.

Our findings indicate that Dop1a titrates PL abundance in response to nutritional availability and licenses cell-cycle entry when sufficient PL is available. In the absence of Dop1a, cells undergo context-unmet cell division even when mTOR signaling is pharmacologically inhibited by Torin1, Torin2, or rapamycin. Importantly, Dop1a deficiency elicits developmental phenotypes in mice and patients even under conditions without overt nutrient deprivation. This raises the question of whether Dop1a-dependent regulation of PL homeostasis is restricted to starvation-induced mTOR inhibition. mTOR signaling is not regulated solely by nutritional availability but is also influenced by multiple developmental and cellular cues, including growth factor signaling, intracellular energy status, cell-cycle progression, and tissue-specific differentiation programs. In the developing brain, mTOR activity is tightly controlled to coordinate anabolic growth with appropriate neuronal proliferation and maturation. Dysregulation of this pathway is well known to result in abnormal brain growth and NDDs.^39^^,^^40^^,^^41^

Our initial analyses in mice revealed an unexpected role for Dop1a in PL metabolism and cellular homeostasis. This prompted us to explore potential clinical relevance, which led to the identification of a patient carrying the R355C substitution in *DOP1A*. The human genetic finding then motivated the generation of a corresponding R355C knock-in mouse line and the establishment of MEFs carrying this mutation. These models enabled us to evaluate the pathogenic potential of the human variant under controlled experimental conditions and to interrogate mechanistic questions that cannot be addressed in patient samples alone.

Genome sequencing of undiagnosed NDD patients identified pathogenic or likely pathogenic *DOP1A* variants in eleven individuals from seven families. Several lines of evidence support the pathogenic relevance of these variants, including intolerance of DOP1A to truncating mutations, the predominance of truncating *de novo* variants, and the loss of function effect of the R355C missense mutation. Patients shared overlapping clinical features such as neurodevelopmental delay, dysmorphic traits, skin abnormalities, and language impairment.

These patient findings prompted us to investigate Dop1a function in neurodevelopment using mouse models. *Dop1a* KO mice exhibited abnormal behaviors in open-field, wire-hang, and three-chamber social interaction tests, corroborating the functional relevance of *Dop1a* in neural development. Insufficient *Dop1a* expression led to cellular overproduction both *in vitro* and *in vivo*. Because cellular composition of the brain is a key determinant of neurodevelopmental disease pathogenesis,^42^^,^^43^^,^^44^ neuronal overgrowth is likely to contribute to aberrant neurobehavioral development, although further studies are required to define the precise pathogenic mechanisms in humans.

In summary, Dop1a functions under the control of LPA-mTOR signaling to lower PL abundance via targeting Agpat2 when LPA availability is limited. NPC targeting of Dop1a restricts CDK2 nuclear accumulation and coordinates PL availability with cell-cycle progression. Proper Dop1a function is therefore essential for normal neurodevelopment, highlighting the importance of regulatory mechanisms governing nuclear PL metabolism during brain development.

### Limitations of the study

This study has several limitations. First, although pathogenic *DOP1A* variants were identified in patients, PL abundance or composition was not directly assessed in patient-derived samples, leaving the contribution of altered PL metabolism to human disease unresolved. Second, while Agpat2 was identified as a downstream effector of Dop1a, the precise molecular mechanism by which Dop1a suppresses Agpat2 activity remains unclear. Third, heterozygous *Dop1a* loss did not markedly affect cell growth in MEFs, whereas heterozygous *DOP1A* variants cause clinical phenotypes in humans, suggesting the involvement of additional genetic or context-dependent modifiers that were not addressed in this study.

## Resource availability

### Lead contact

Further information and requests for resources and reagents should be directed to and will be fulfilled by the lead contact, Masaki Mori (mori-ma@ncchd.go.jp).

### Materials availability

All unique reagents generated in this study are available from the lead contact upon reasonable request.

### Data and code availability

The scRNA-seq data generated in this study have been deposited in the DNA Data Bank of Japan (DDBJ) database under the BioProject accession number PRJDB40458 and experiment accession number E-GEAD-1221. Additional datasets are available from the lead contact upon reasonable request.

## Acknowledgments

We are grateful to all members of the Department of Pediatric Physiology at the National Center for Child Health and Development for their valuable discussions and generous support. The Central Research Laboratory at Shiga University of Medical Science (SUMS) supported this study. We would like to thank Keisuke Nimura for the critical reading. M.M. is supported by research grants from the Japan Epilepsy Research Foundation (JERF), Japan Brain Foundation, Takeda Science Foundation, the Japan Spina Bifida & Hydrocephalus Research Foundation, Kawano Masanori Memorial Public Interest Incorporated Foundation for Promotion of Pediatrics, Grants-in-Aid for Scientific Research for Young Scientists from the Japan Intractable Diseases (Nanbyo) Research Foundation, and Japanese Rare Disease Models & Mechanisms Network (J-RDMM). Takeda Science Foundation supported T.M. and N.M. This study was supported by JSPS KAKENHI grant nos. JP22K19499, JP22H05643, JP24H01417, and JP24K02247 (to M.M.), JP23H02829 (to S.M.), JP21K07869 (to E.K.), JP23H02877 (to T.M.), JP24K02230 (to N.M.), by AMED under grant nos. JP22gm6710009755 (to M.M.), P25ek0109674, JP25ek0109760, JP25ek0109617, JP25ek0109648, and JP25ek0109677 (to N.M.), and by Japan Health Research Promotion Bureau (JH) 2021-B-01 (to M.M.).

## Author contributions

H.A. performed biochemical and animal anlayses; A.T. performed mouse behavioral experiments and histological experiments; S.M. organized clinical analysis and anayzed clinical data; S.O. performed PL, biochemical, and animal and histological analyses; I.I., A.O., A.S., and H.K. assisted biochemical and animal experiments; Y.S., Y.N., and R.S. performed human iPSC-based experiments; I.T. generated genetically modified mouse strains; E.K. and T.M. assisted clinical data collection and analysis; K.H. and K.O. performed DOP1A protein structural analysis; E.N. and T.S. performed genetic and clinical analysis with patient 1; M.S. performed scRNA-seq analysis; Y.F. generated genetically modified mouse strains; M.U., M.Q., C.R., A.G.K., M.N.K., and M.A. performed genetic and clinical analysis with patients 4 and 5; E.H.G., T.R., A.P.A.S., and M.S. performed genetic and clinical analysis with patients 2 and 3; M.A.A., E.A., R.M.B., and N.A.M.A. performed genetic and clinical analysis with patients 6, 7, 8, and 9; S.B., A.P., C.D.C., S.M., B.C., and P.Y. performed genetic and clinical analysis with patients 10 and 11; S.-y.M. assisted PL analyses; A.K. performed neuropathological evaluations; M.K. performed genetic and clinical analysis with patient 1; Y.M. analyzed data and provided feedback on the manuscript; N.M. supervised the clinical and genetic studies and manuscript writing; M.M. conceived and supervised the study and contributed to experimental work, data analysis, and manuscript writing. All authors reviewed and approved the final manuscript.

## Declaration of interests

The authors declare no competing interests.

## STAR★Methods

### Key resources table

**Table:** 

REAGENT or RESOURCE	SOURCE	IDENTIFIER
**Antibodies**		

DOP1A	Atlas Antibodies	HPA027904
DOP1B/DOPEY2	Atlas Antibodies	HPA072065
GM130	BD Biosciences	610823
Lamin B1	ProteinTech	66095-1-Ig
Nup153	Abcam	ab24700
Agpat2	CST	#14937
PLD1	CST	#3832
BSCL2/Seipin	CST	#23846
GPAT1	Millipore	ABS764
PCYT1A	Bethyl	A304-559A-M
CDK2	CST	#18048S
Ki67	CST	#12202
p27	CST	#3698
PCNT	Abcam	ab4448
anti-rabbit-Alexa Fluor 488	Thermo Fisher Scientific	A11008
anti-mouse-Alexa Fluor 546	Thermo Fisher Scientific	A11030
anti-mouse-Alexa Fluor 647	Thermo Fisher Scientific	A21235
Calr	Abcam	ab196159
PDI	CST	45596S
alpha-tubulin	CST	#3873
Pol2	Covance	MMS-126R-200
Gapdh	CST	#5174
anti-mouse IgG	Thermo Fisher Scientific	#32430
anti-rabbit IgG	Thermo Fisher Scientific	#32460

**Chemicals, peptides, and recombinant proteins**		

Torin1	Tocris Bioscience	No. 4247
Torin2	Tocris Bioscience	No. 4248
Rapamycin	Fujifilm Wako	550–83271
retinoic acid	Tocris Bioscience	No. 0695
Forskolin	Tocris Bioscience	No. 1099
sodium arsenite	Merck	S7400
Zeocin	Thermo Fisher Scientific	R25001
Ki16425	Merck	SML0971
LPA	Fujifilm Wako	62215
4EGI-1	Selleck Chemicals	S7369
Bafilomycin A1	Selleck Chemicals	S1413

**Critical commercial assays**		

Total Phosphatidic Acid Assay Kit	Cell Biolabs	MET-5019
Phosphatidylcholine Assay Kit	Cell Biolabs	STA-600

**Deposited data**		

scRNA-seq data (this study)	DDBJ	PRJDB40458

**Experimental models: Cell lines**		

Neuro2a cells	JCRB Cell Bank	RRID: CVCL_0470
NIH3T3cells	JCRB Cell Bank	RRID: CVCL_0594

**Experimental models: Organisms/strains**		

C57BL6/N mice	CLEA Japan, Inc.	RRID: MGI:7466658

**Oligonucleotides**		

See Table S3 for primers used in the gene expression analysis.	NA	NA
gRNA to generate Dop1a KO, 5′-AGAAGAAAGCACTCTCATGCAGG-3′	This study	N.A.
Genotyping primers for Dop1a KO: forward 5′-CTGCGTTTCAGATGGGTACA-3′ and reverse 5′-CCAGCTGCACACCT GTAACT-3'.	This study	N.A.
gRNA to generate Dop1a-R355C, 5′-AAACTGATTAAAATACGAAA-3′	This study	N.A.
ssDNA to generate Dop1a-R355C: 5′-gaaggccatggtggggatcttacaag tgaatggatttggagaagaaagcactct catgcaggatctaaaaccttttTgtatAttaa tcagtttattggacaaacctgaactaggtaa tatttggtgctgttccggaacataaggtga catg-3′	This study	N.A.
Genotyping primers for Dop1a-R355C: forward 5′-GCAGTGTGCTTTCTCCCT GGCGC-3′ and reverse 5′-GCTGA CATAAAGGCCAGCTGCACACC-3’.	This study	N.A.

**Software and algorithms**		

GraphPad Prism v.9.0	Dotmatics	N.A.
ImageJ	NIH	N.A.
R	The R Foundation	N.A.
Seurat v4	Satija lab	N.A.

### Experimental model and study participant details

#### Animal experiments

All animal experiments were approved by the Institutional Animal Care and Use Committees of Shiga University of Medical Science (approval number: [2020-9-1(H1)]), the National Cerebral and Cardiovascular Center (approval number: [20075]), and the National Center for Child Health and Development (approval number: [A2024-005]). All experiments were conducted in accordance with the relevant institutional guidelines and national regulations.

#### Establishment of *Dop1a* mutant mice

##### Reagents for genome editing

The *Dop1a* mutant mouse strains were generated using CRISPR-Cas9 technology as described previously.^45^ The gRNA was designed using CRISPRdirect (https://crispr.dbcls.jp/). The sequence of the gRNA is 5′-AGAAGAAAGCACTCTCATGCAGG-3’. The gRNA sequence was cloned into the gRNA cloning vector (Addgene Plasmid ID #41824). For gRNA synthesis, the T7 RNA polymerase recognition site was attached to the gRNA sequence via the polymerase chain reaction (PCR). The PCR products were purified and used as the template for *in vitro* RNA synthesis using the mMessage mMachine T7 Transcription Kit (Life Technologies). The synthesized gRNA was purified using MEGAclear (Ambion). Recombinant Cas9 protein was purchased (GeneArt Platinum Cas9 Nuclease, Thermo Scientific, B25642).

##### Microinjection

Mouse zygotes were obtained via *in vitro* fertilization (IVF) of WT C57BL6/N (RRID: MGI:7466658) gametes. gRNA (100 ng/mL) and Cas9 protein (30 ng/mL, TrueCut Cas9 Protein v2, Life Technologies, MA) were mixed and microinjected into the pro-nuclei and cytoplasm of zygotes. The injected embryos were incubated at 37°C until they were transferred into pseudo-pregnant females at the two-cell stage.

##### Genotyping and breeding

Genomic DNA was extracted from the tail tips of pups, and the genomic sequence around the gRNA target site was PCR-amplified using the following primers: forward 5′-CTGCGTTTCAGATGGGTACA-3′ and reverse 5′-CCAGCTGCACACCTGTAACT-3'. The strain with a 94-nucleotide insertion and the other strain with a 69-nucleotide deletion were analyzed, yielding consistent results. The findings with the 94-nucleotide insertion were presented in this study.

To generate R355C mice, CRISPR-Cas9-mediated production of mutant mice was performed as described previously.^46^ Briefly, *Dop1a* R355C knockin mice were generated by introducing the gRNA/CAS9 protein solution and single-stranded DNA (ssDNA; IDT, IA, USA) into fertilized eggs with an electroporator (NEPA21, Nepagene, Chiba, Japan). A search for gRNA and off-target sequences was performed using CRISPRdirect software (http://crispr.dbcls.jp/). Screening of the obtained mutant mice was performed by direct sequencing following PCR. The gRNA used was 5′-AAACTGATTAAAATACGAAA-3′ for the ninth exon of *Dop1a*. The ssDNA used was:

5′-gaaggccatggtggggatcttacaagtgaatggatttggagaagaaagcactctcatgcaggatctaaaaccttttTgtatAttaa tcagtttattggacaaacctgaactaggtaatatttggtgctgttccggaacataaggtgacatg-3′ for R355C substitution (cgt→Tgt) with the AseI site (5′-attaat-3′) introduced via silent mutation (att→atA). Genotyping of the R355C mice was determined by PCR using KOD FX *Neo* DNA polymerase (KFX-201, Toyobo, Japan) with the following primers: Dop1a-TF (#241) 5′-GCAGTGTGCTTTCTCCCTGGCGC-3′ and Dop1a-TR (#242) 5′-GCTGACATAAAGGCCAGCTGCACACC-3’. To distinguish the alleles, the amplified PCR products were subsequently subjected to restriction digestion with AseI (319–02541, Nippon Gene, Japan). This enzyme specifically cleaves the mutant allele due to an AseI site (5′-attaat-3′) introduced via a silent mutation (att→atA).

#### Human analysis

##### Study participants

This study was approved by the Committee for Ethical Issues at Yokohama City University School of Medicine and Showa University School of Medicine, the institutional review boards at Maastricht University Medical Center, the Ethikkommission Nordewest- and Zentralschweiz (2019-01660) and the Khalifa Gul Nawaz Teaching Hospital, Bannu, King Saud University (IRB_Approval on research project No. E−18-2838), and Taibah University (MLT-2019-07) and all adhered to the tenets of the Declaration of Helsinki. The principal Institutional Review Board approval was obtained from the Ethics Committee of the Strasbourg University Hospital (CCPPRB, patient 10) and Nantes University Hospital (patient 11). All participants in this study or their legal guardians were informed about the purpose of this research before they signed a written informed consent form.

##### Genomic analysis

For patient 1, trio-based whole-exome sequencing was performed using genomic DNA extracted from leukocytes at Yokohama City University as previously described.^47^ Detected variants were confirmed by Sanger sequencing.

For patient 2, the *DOP1A* variant was detected by routine WES diagnostics and variant calling using a parent-offspring (trio) approach. Briefly, the exome was captured using the Agilent SureSelectXT Human All Exon V5 library prep kit (Agilent Technologies, Santa Clara, CA, USA). Exome libraries were sequenced on an Illumina HiSeq 2000 instrument (Illumina, San Diego, CA, USA) with 101 bp paired-end reads at a median coverage of 75× at the BGI Europe facilities (BGI, Copenhagen, Denmark). Sequence reads were aligned to the hg19 reference genome using Burrows-Wheeler Alignment (BWA, Li and Durbin, 2009), and variants were subsequently called by the Genome Analysis Toolkit (GATK, https://gatk.broadinstitute.org/hc/en-us) unified genotyper, version 3.2–2, and annotated using a custom-built diagnostic annotation pipeline.

For patient 3, trio-based whole-exome sequencing was performed. In brief, exons were captured using an Agilent SureSelect Human All Exon 50 Mb Kit (Agilent Technologies, Santa Clara, CA, USA). Sequencing was performed using an Illumina HiSeq platform (San Diego, CA, USA). Read mapping and variant calling were done using BWA^48^ and GATK, respectively. Variants were prioritized based on the following criteria: frequency (<5% dbSNP, <1% in-house database of >20,000 exomes), nucleotide and amino acid conservation (based on alignments), relation of the gene to disease (per family), and inheritance pattern. Annotation of variants was done using in-house pipelines.

For patients 4 and 5, saliva samples were collected from affected and healthy individuals using the Oragene-DNA self-collection kits (DNA Genotek Inc., Ottawa, Canada). Then, genomic DNA was extracted from saliva using the prepI-L2P protocol, following the manufacturer’s instructions (DNA Genotek Inc., Canada). Whole-exome sequencing of the proband was performed using the Twist human core exome kit (Twist Bioscience, San Francisco, USA) through the Illumina NovaSeq 6000 instrument at CeGat GmbH (Tübingen, Germany). Exome data were analyzed using an already established informatics pipeline, as previously described.^49^ Briefly, raw sequences were mapped to the Human Genome Reference sequence (hg19/GRCh37) using BWA mem (v0.7.17). Then, Picard (v2.14.0-SNAPSHOT) and GATK (v4.1.4.1) were used for further processing and variant calling. ANNOVAR^50^ was used to annotate variants. Gene annotations were added with in-house scripts. The resulting variants were then prioritized based on their quality, allelic frequency in databases, molecular profile, and finally, according to compatible patterns of inheritance and their presence within the list of genes currently associated with ID. AutoMap was used to identify the runs of homozygosity from the exome data.^51^

For patients 6–9, genomic DNA was extracted from whole blood samples collected in EDTA tubes from all Family 5 members, including parents, patients, and unaffected siblings. Duo whole-exome sequencing was carried out for patients 6 and 8. Briefly, the DNA libraries were prepared and sequenced using the SureSelect V6-Post (Agilent, Santa Clara, CA, USA) and the NovaSeq 6000 system (Illumina, San Diego, CA, USA), respectively. The GATK was used to map the genome sequencing and generate high-quality variant calls. ACMG guidelines were followed to classify variants in known and candidate genes. Allele frequencies were verified using the gnomAD. Sanger sequencing for confirmation was performed for all family members.

For patient 10, trio-based whole genome sequencing was performed using genomic DNA extracted from leukocytes at Strasbourg Hospital as previously described.^52^ Detected variants were confirmed by Sanger sequencing.

For patient 11, whole-exome sequencing was performed at Nantes University Hospital.

### Method details

#### Cell culture

Neuro2a cells (RRID: CVCL_0470) were cultured in Eagle’s minimal essential medium supplemented with non-essential amino acids, 10% fetal bovine serum (FBS), and penicillin/streptomycin. NIH3T3cells (RRID: CVCL_0594) were cultured in DMEM containing 10% FBS and penicillin/streptomycin.

Mouse primary neurons were isolated from the cerebral cortices of WT and *Dop1a* KO fetuses on E15.5. The cerebral cortices were washed with chilled HBSS and then incubated in a solution containing trypsin and DNase. Tissues were gently dissociated by pipetting and filtered with a strainer. Cells were plated at 5 × 10^6^ cells/ml in the Neurobasal Medium (21103049, ThermoFisher Scientific). AraC was added to the culture media the next day at 10 μM.

Mouse embryonic fibroblasts (MEFs) were derived from WT and R355C mice on E13.5. Briefly, bodies were minced with a blade in the presence of trypsin and then incubated at 37°C for 30–45 min. Tissues were further dissociated by gentle pipetting. Trypsin was inactivated by adding serum-containing culture media. The cell suspensions were put into the culture in the presence of culture media.

In the knockdown experiments, Lipofectamine RNAiMAX (Invitrogen) was used to transfect Neuro2a and NIH3T3cells with the siRNA duplexes at 20 nM: human DOP1A_1 (Sigma-Aldrich, siRNA ID SASI_Hs01_00040947), human DOP1A_2 (siRNA ID SASI_Hs01_00040948), mouse Dop1a siRNA_1 (siRNA ID SASI_Mm02_00347223), mouse Dop1a siRNA_2 (siRNA ID SASI_Mm02_00347225), mouse Nup153 siRNA_1 (siRNA ID SASI_Mm01_00133193), mouse Nup153 siRNA_2 (siRNA ID SASI_Mm02_00133194), mouse Agpat2 siRNA_1 (siRNA ID SASI_Mm01_00175545), mouse Agpat2 siRNA_2 (siRNA ID SASI_Mm02_00175546), and siRNA negative control (AM4611, Thermo Fisher Scientific, MA, USA). The knockdown efficiency was assessed 48 h after transfection by real-time qPCR and western blot analyses.

Cellular growth was assessed by counting the number of cells 72 h after transfection. The phase-contrast images of the cells were taken using an EVOS FL microscope (Thermo Fisher Scientific).

The following compounds were added to the culture medium at the indicated concentrations: Torin1 (250 nM, Tocris Bioscience, UK), Torin2 (200 nM, Tocris Bioscience, UK), rapamycin (10 μM, Fujifilm Wako, Japan), retinoic acid (20 nM, Tocris Bioscience, UK), forskolin (100 μM, Fujifilm Wako, Japan), sodium arsenite (0.5 mM, Merck, Germany, USA), zeocin (40 μg/mL, Thermo Fisher Scientific) and Ki16425 (10 μM, Merck, Germany, USA).

For visualization of cellular lipids, cells were incubated with Bodipy 493/503 (D3922, Thermo Fisher Scientific) or Bodipy fatty acid (D3835, Thermo Fisher Scientific) for 30 min to stain neutral lipids or fatty acids, respectively.

The response to LPA was assessed in serum-starved cells by adding LPA (1-Oleoyl lysophosphatidic acid, 62215, Fujifilm Wako, Japan) at 10 μM with daily replenishment.

4EGI-1 (S7369, Selleck Chemicals, HOU) was added at a concentration of 5 μM. Bafilomycin A1 (S1413, Selleck Chemicals) was added at a concentration of 250 nM.

#### Human induced pluripotent stem cells (iPSCs)

Human iPS cells established previously^53^ were differentiated into neural stem cells (NSCs) using PSC Neural Induction Medium (Thermo Fisher Scientific, MA) and maintained on 1% Geltrex Flex LDEV-Free hESC-Qualified Reduced Growth Factor Matrix (Thermo Fisher Scientific, MA) in NSC expansion medium (Neurobasal: DMEM/F12 = 1:1 supplemented with NSC supplements). For RNAi experiments, NSCs were seeded onto 1% Geltrex-coated 24-well plates at 6 × 10^4^ cells per well in Neurobasal medium. After 24 h, cells were transfected with 50 nM siRNAs targeting DOP1A (SASI_Hs01_00040947 and SASI_Hs01_00040948; Sigma-Aldrich). The culture medium was replaced with fresh medium 16 h after transfection, and cells were subsequently cultured for 48 h in the presence of Torin1 (250 nM) or DMSO, followed by imaging and cell quantification.

#### Gene expression analysis

The total RNA was extracted using TRIzol reagent (Thermo Fisher Scientific, MA) from cultured cells, following the manufacturer’s instructions. The extracted RNA was quantified using a NanoDrop Lite Spectrophotometer (Thermo Fisher Scientific, MA) and reverse transcribed using a High-Capacity RNA-to-cDNA Kit (Applied Biosystems) and a PCR Thermal Cycler Dice (Takara Bio, Japan) according to the manufacturer’s instructions. qPCR was performed using a LightCycler 480 SYBR Green I Master Kit on a LightCycler 480 instrument (Roche, Switzerland) using the reverse-transcribed cDNA as a template. The specificity and quality of the qPCR amplification were assessed through melting curve analysis. The data were normalized to mouse *Polr2a*, *Gapdh*, or human *GAPDH* as indicated in the Figure legends. The sequences of the primers are shown in Table S3.

#### Single-cell RNA-seq

The study used male C57BL/6N mice (RRID: MGI:7466658) on P7 and P14. Single cells from the mouse cerebral cortex were isolated using the gentleMACS Octo Dissociator with Heaters (Miltenyi Biotec, Germany), following the manufacturer’s instructions. The resulting single-cell suspension was filtered through a 70 μm cell strainer to remove undissociated tissue fragments. Single-cell RNA sequencing was performed using the 10X Chromium system (10X Genomics, CA, USA) to enable reverse transcription at the single-cell resolution. The isolated single cells were loaded onto a 10X Genomics chip, where cell lysis, barcoding, and cDNA synthesis co-occurred. The prepared library was then run on a NextSeq 500 sequencer (Illumina, San Diego, CA, USA) according to the manufacturer’s instructions. The raw sequencing data were processed using the Cell Ranger software (10X Genomics) to generate gene expression matrices and cell barcode files. The resulting count data were further analyzed using the Seurat package in R (https://satijalab.org/seurat/)^54^ to identify cell clusters and differentially expressed genes.

#### Immunofluorescence analysis

Cells were fixed with 4% paraformaldehyde (PFA) for 10 min at room temperature (RT), permeabilized with 0.1% Triton X-100 for 2 min, blocked with 2% FBS at RT and incubated with antibody against DOP1A (Atlas Antibodies, HPA027904, 1:100), DOP1B/DOPEY2 (Atlas Antibodies, HPA072065, 1:100), GM130 (BD Biosciences, 610823, 1:400), PDI (CST, #45596S, 1:200), Lamin B1 (ProteinTech, 66095-1-Ig, 1:400), Nup153 (Abcam, ab24700. 1:400), Agpat2 (CST, #14937, 1:200), PLD1 (CST, #3832, 1:200), BSCL2/Seipin (CST, #23846, 1:200), GPAT1 (Millipore, ABS764, 1:200), PCYT1A (Bethyl, A304-559A-M, 1:100), CDK2 (CST, #18048S, 1:200), Ki67 (CST, #12202, 1:500), p27 (CST, #3698, 1:200), and PCNT (Abcam, ab4448, 1:400) at 4 °C overnight. After washing with phosphate-buffered saline (PBS) for 10 min 3 times, cells were incubated with appropriate secondary antibodies anti-rabbit-Alexa Fluor 488 (A11008, Thermo Fisher Scientific), anti-mouse-Alexa Fluor 546 (A11030), or anti-mouse-Alexa Fluor 647 (A21235) at RT for 1 h. Slides were mounted with Prolong Diamond with DAPI (P36971, Thermo Fisher Scientific). Images were taken using a confocal microscope, TCS SP8 (Leica, Germany) and FLUOVIEW FV3000 (Olympus, Japan). Colocalization was quantified using the Coloc2 plugin in Fiji (NIH ImageJ).

#### SDS-PAGE and western blot analysis

The cells were harvested in RIPA lysis buffer containing 25 mM Tris-HCl (pH 7.6), 150 mM NaCl, 1% NP-40, 1% sodium deoxycholate, and 0.1% SDS. For the collection of the NM-containing nuclear fraction, Neuro2a cells were treated with Torin1 for 72 h. Following treatment, cells were harvested and subjected to subcellular fractionation using the NE-PER Nuclear and Cytoplasmic Extraction Reagents (Thermo Fisher Scientific, Cat#78833) according to the manufacturer’s instructions. Mouse tissues were crushed using a homogenizer (μT-12, Taitec, Japan) in RIPA buffer, and the lysates were centrifuged at 20,400 ×g for at least 5 min to remove debris. Then, 5x Laemmli sample buffer was added to the lysates, followed by boiling at 95°C for 2 min. The protein samples (10 μg per lane) were separated by SDS-PAGE and transferred to a Hybond-P PVDF membrane (GE10600023, GE Healthcare). Western blotting was performed using the following antibodies: DOP1A (Atlas Antibodies, HPA027904, 1:100), Lamin B1 (ProteinTech, 66095-1-Ig, 1:2000), Nup153 (Abcam, ab24700. 1:2000), Agpat2 (CST, #14937, 1:1000), Calr (Abcam, ab196159, 1:1000), PDI (CST, #45596S, 1:1000), α-tubulin (CST, #3873, 1:2000), Pol2 (Covance, MMS-126R-200, 1:1000), and Gapdh (CST, #5174, 1:2000). Secondary antibodies conjugated with horseradish peroxidase (#32430 anti-mouse IgG, #32460 anti-rabbit IgG, Thermo Fisher Scientific) were used at 1:1000 dilution. The immunoreactive bands were detected using Chemi-Lumi One Super (#02230, Nacalai Tesque, Japan) or Chemi-Lumi One Ultra (#11644, Nacalai Tesque, Japan).

#### Immunoprecipitation

Cells were lysed with NETN buffer (100 mM NaCl, 20 mM Tris-Cl [pH 8.0], 0.5 mM EDTA, 0.5% Nonidet P-40). After centrifugation at 20,000 x g at 4 °C for 5 min, the lysates were pretreated with Protein G Sepharose 4 Fast Flow (GE17-0618-05, Cytiva, MA, USA) for 1 h to remove background binding to the beads and then incubated with antibodies at 4 °C overnight. The protein-antibody complexes were incubated with Protein G Sepharose 4 Fast Flow at 4 °C for 1 h. The beads were washed three times with NETN 200 buffer (200 mM NaCl, 20 mM Tris-Cl [pH 8.0], 0.5 mM EDTA, and 0.5% Nonidet P-40). The sample buffer was added and incubated at 95°C for 5 min. After centrifugation at 20,000 x g for 1 min, the supernatants were collected for western blot analysis.

#### Mouse histological analysis

For histological analysis of the brains, male mice were systemically fixed with 4% PFA on P7, followed by dissection of the organs. The brain was dissected to create a slice that showed the hippocampus. The dissected organs were post-fixed with 4% PFA for 3 h, followed by sectioning at a thickness of 3 μm. For the immunofluorescence analysis, the PFA-fixed sections were permeabilized with 0.1% Triton X-100 for 2 min and blocked with 2% FBS at RT for 60 min. The samples were incubated with the primary antibody against Dop1a at 4 °C overnight. After washing with PBS, the tissues were incubated with the secondary antibody anti-mouse-Alexa Fluor 488 (A28175, 1:1,000, Invitrogen). After washing with PBS, the tissues were mounted with Prolong Diamond with DAPI (P36971, Invitrogen). Images were taken using a confocal microscope TCS SP8 (Leica).

#### Metabolome analysis

We conducted metabolome analysis of cellular samples using LC-MS/MS with the Nexera UHPLC system and LCMS-8050. We normalized the quantitative data to the number of cells. For the high-performance liquid chromatography (HPLC), we utilized acetonitrile (Kanto), formic acid (FUJIFILM Wako Pure Chemical Corporation), and five mol/L hydrochloric acid for volumetric analysis (FUJIFILM Wako Pure Chemical Corporation). We used 2-isopropylmalic acid (Sigma-Aldrich) as the internal standard (IS). To prepare the samples, we washed cells cultured on 10 cm plates twice with PBS and scraped them into 2 mL LC-grade methanol. We added five μL of IS solution to 25 μL of the samples, followed by 20 μL of 0.01 mol/L hydrochloric acid and then 50 μL of acetonitrile to agitate the solution. We centrifuged the samples at 10,000 rpm at RT for 5 min and subjected the supernatants to MS analysis. We used the Discovery HS F5-3 (2.1 mm I.D. ×150 mm, three μm, Sigma-Aldrich) column and a mobile phase consisting of 0.1% formic acid in water (mobile phase A) and 0.1% formic acid in acetonitrile (mobile phase B). The gradient program was as follows.

**Table:** 

Time (min)	0	2	5	11	15	20	20.1	25
Mobile Phase B (%)	0	0	25	35	95	95	0	0

The flow rate was 0.25 mL/min, the injection volume was three μL, and the column oven temperature was 40°C. The ionization mode used for MS analysis was electrospray ionization (ESI), both in positive and negative modes.

We analyzed the data using LabSolutions (Shimadzu Corporation). The peak area was measured, and the IS ratio was calculated by comparing the peak area of each compound with that of the IS. We obtained the IS average values for the test and control groups using IS ratios. Finally, we compared the metabolite variation between the two groups and the average values of the IS.

#### Phospholipid analysis

PL-MS was performed using the following chemicals: PA, PS, and PG (Sigma-Aldrich, St. Louis, USA); PE, chloroform, and 28% ammonia water (Fujifilm Wako Pure Chemical Corporation, Japan); and PC and PI (Nacalai Tesque, Japan). Dichloromethane and methanol were purchased from Kanto Chemical Co., Inc. (Japan), and water was purified using a Milli-Q Integral MT5 system (Millipore, Boston). A mixture of chloroform/methanol (4/1, v/v) and a 90% methanol solution containing 0.1% ammonia was prepared for the experiment. A standard solution was prepared by dissolving PA, PS, PE, PC, PI, and PG in a chloroform/methanol mixture (4:1, v/v) and diluting it to concentrations of 50, 100, 250, 500, 1000, and 2500 ng/mL. Cell samples were collected in 2 mL of methanol for sample preparation. Cerebral cortex tissues from male mice at P14 were minced and measured before being added to chloroform. After centrifugation and transfer of the organic layer, the residue was reconstituted with a 4:1 (v/v) mixture of dichloromethane and methanol to prepare analytical samples. Chromatographic separation of phospholipids was achieved on an Inertsil SIL-100A column with a Nexera UHPLC system. Detection was performed using a Triple Quad 5500 mass spectrometer with electrospray ionization. We normalized the quantitative data to the number of cells. Data were analyzed using Analyst 1.7 and MetaboAnalyst 5.0. Quantitation plots of each phospholipid peak area versus the concentration of PL in the standard solution were constructed, and the composition ratio of the sum of the alkyl chain lengths and the sum of the unsaturation degrees of the fatty acids was calculated. Data were normalized to the number of nuclei to determine the abundance of PLs per cell. For heatmap visualization, the square ratios were calculated into z-scores. Metabolite set enrichment assay (MSEA) was performed using MetaboAnalyst 6.0.^55^ Phosphatidic acid and phosphatidylcholine were measured using the Total Phosphatidic Acid Assay Kit (MET-5019, Cell Biolabs) and the Phosphatidylcholine Assay Kit (STA-600, Cell Biolabs), respectively, according to the manufacturer’s instructions.

#### Mouse behavioral analysis

##### Open field testing

The open field test was conducted in a circular arena with a diameter of 38 cm, recorded using an overhead camera, and tracked and analyzed using the ANY-maze video tracking software (Muromachi, Japan). Before testing, the arena was cleaned with a 70% ethanol and water solution. Male mice (*n* = 13, 14) aged 10–12 weeks were introduced to the arena and allowed to explore for 10 min while being tracked. The total distance traveled, the number of entries to the central area, and the time spent within the central circle were measured.

##### Wire-hang test

The wire hang test assessed male mice’s grip strength and motor coordination at 10–12 weeks old (*n* = 16, 19). The test was conducted using a wire mesh placed 30 cm above a soft surface to prevent injury to the mice. Mice were trained for two consecutive days before testing to ensure they could grip the wires and hang for at least 60 s. Each mouse was placed on the wire mesh for the wire hang test and allowed to grip the wires with their forelimbs. The lid was then gently turned upside down, allowing the mouse to hang upside down from the wires. The latency to fall from the wire mesh was recorded for each trial, with a maximum cut-off time of 180 s. Each mouse was tested three times, with a 60-s interval between trials.

##### Three-chamber sociability test

The three-chamber sociability test used a 60 × 40 cm^2^ sociability apparatus (SC-03M, Muromachi, Japan). To begin, male mice (*n* = 15) aged 10–12 weeks were habituated to the empty arena for 10 min. Subsequently, mice were confined to the center chamber, and a sex- and age-matched adult, C57BL/6N mouse, was placed in a small cage in one chamber (social chamber) while the other cage was kept empty (non-social chamber). Mice were then allowed to travel between chambers for 10 min, and the movement of mice was tracked using the ANY-maze video tracking software (Muromachi, Japan). For the social novelty phase, the subject mouse was placed back into the middle chamber while a novel mouse of the same strain was placed in the previously empty cup. The location and behavior of the subject mouse were recorded for an additional 10 min. Before each test, the arenas were disinfected using a 70% ethanol and water solution.

#### Structural modeling

We performed a structural modeling of the DOP1A by AlphaFold2^56^ for the structural considerations of the missense variations in this study. In the calculated model structure using AlphaFold2, the prediction reliability is denoted as the predicted local distance difference test (pLDDT), with values ranging from 0 to 100. Regions with very high confidence (pLDDT >90) are colored in blue, high confidence (90 > pLDDT >70) in cyan, low confidence (70 > pLDDT >50) in yellow, and very low confidence (pLDDT <50) in orange. FoldX^57^^,^^58^ was used to calculate the free energy changes due to the missense variations. Structural figures were drawn using PyMOL (Schrödinger, Inc.).

### Quantification and statistical analysis

All the biological experiments were repeated at least three times to assess reproducibility. The presented data were collected from biologically independent samples. Statistical analyses were performed using GraphPad Prism v.9.0 (GraphPad, MA). All data are represented as mean ± SEM unless otherwise specified. Unpaired Student’s *t*-tests were used for two-group comparisons. For multiple comparisons, data were initially analyzed using a one-way analysis of variance (ANOVA) to determine whether there were significant differences among the groups. Post-hoc comparisons were conducted using Tukey’s multiple comparisons test to identify specific group differences. The statistical parameters and mouse numbers used per experiment are specified in the figure legends. Statistical methods were not used to predetermine the sample size. *p* < 0.05 was considered to be significant throughout the study.

## Supplemental information

Document S1. Figures S1–S7

Table S1. Clinical information, related to Figure 6

Table S2. Evaluation of DOP1A variants, related to Figure 6

Table S3. Sequences of primers used in the gene expression analyses, related to Figures 2, 5, S1, S3, and S6

## References

[1] Hetzer M.W. (2010). The Nuclear Envelope. Cold Spring Harb. Perspect. Biol..

[2] De Magistris P., Antonin W. (2018). The Dynamic Nature of the Nuclear Envelope. Curr. Biol..

[3] Schooley A., Vollmer B., Antonin W. (2012). Building a nuclear envelope at the end of mitosis: coordinating membrane reorganization, nuclear pore complex assembly, and chromatin de-condensation. Chromosoma.

[4] Antonin W., Ellenberg J., Dultz E. (2008). Nuclear pore complex assembly through the cell cycle: regulation and membrane organization. FEBS Lett..

[5] Webster M., Witkin K.L., Cohen-Fix O. (2009). Sizing up the nucleus: nuclear shape, size and nuclear-envelope assembly. J. Cell Sci..

[6] Fagone P., Jackowski S. (2009). Membrane phospholipid synthesis and endoplasmic reticulum function. J. Lipid Res..

[7] Ungricht R., Kutay U. (2015). Establishment of NE asymmetry-targeting of membrane proteins to the inner nuclear membrane. Curr. Opin. Cell Biol..

[8] Vance J.E. (2015). Phospholipid synthesis and transport in mammalian cells. Traffic.

[9] Romanauska A., Köhler A. (2018). The Inner Nuclear Membrane Is a Metabolically Active Territory that Generates Nuclear Lipid Droplets. Cell.

[10] Romanauska A., Köhler A. (2021). Reprogrammed lipid metabolism protects inner nuclear membrane against unsaturated fat. Dev. Cell.

[11] Barbosa A.D., Lim K., Mari M., Edgar J.R., Gal L., Sterk P., Jenkins B.J., Koulman A., Savage D.B., Schuldiner M. (2019). Compartmentalized Synthesis of Triacylglycerol at the Inner Nuclear Membrane Regulates Nuclear Organization. Dev. Cell.

[12] Krahmer N., Guo Y., Wilfling F., Hilger M., Lingrell S., Heger K., Newman H.W., Schmidt-Supprian M., Vance D.E., Mann M. (2011). Phosphatidylcholine synthesis for lipid droplet expansion is mediated by localized activation of CTP:phosphocholine cytidylyltransferase. Cell Metab..

[13] Shindou H., Shimizu T. (2009). Acyl-CoA:lysophospholipid acyltransferases. J. Biol. Chem..

[14] Sołtysik K., Ohsaki Y., Tatematsu T., Cheng J., Maeda A., Morita S.Y., Fujimoto T. (2021). Nuclear lipid droplets form in the inner nuclear membrane in a seipin-independent manner. J. Cell Biol..

[15] Morita S.Y., Ikeda Y. (2022). Regulation of membrane phospholipid biosynthesis in mammalian cells. Biochem. Pharmacol..

[16] Wu Y., Chen K., Xing G., Li L., Ma B., Hu Z., Duan L., Liu X. (2019). Phospholipid remodeling is critical for stem cell pluripotency by facilitating mesenchymal-to-epithelial transition. Sci. Adv..

[17] Yi K., Zhan Q., Wang Q., Tan Y., Fang C., Wang Y., Zhou J., Yang C., Li Y., Kang C. (2021). PTRF/cavin-1 remodels phospholipid metabolism to promote tumor proliferation and suppress immune responses in glioblastoma by stabilizing cPLA2. Neuro Oncol..

[18] Sonkar K., Ayyappan V., Tressler C.M., Adelaja O., Cai R., Cheng M., Glunde K. (2019). Focus on the glycerophosphocholine pathway in choline phospholipid metabolism of cancer. NMR Biomed..

[19] Lo Presti C., Fauvelle F., Jacob M.C., Mondet J., Mossuz P. (2021). The metabolic reprogramming in acute myeloid leukemia patients depends on their genotype and is a prognostic marker. Blood Adv..

[20] Wang B., Tontonoz P. (2019). Phospholipid Remodeling in Physiology and Disease. Annu. Rev. Physiol..

[21] Tsuji T., Morita S.Y., Nakamura Y., Ikeda Y., Kambe T., Terada T. (2021). Alterations in cellular and organellar phospholipid compositions of HepG2 cells during cell growth. Sci. Rep..

[22] Otsuka S., Tempkin J.O.B., Zhang W., Politi A.Z., Rybina A., Hossain M.J., Kueblbeck M., Callegari A., Koch B., Morero N.R. (2023). A quantitative map of nuclear pore assembly reveals two distinct mechanisms. Nature.

[23] Dultz E., Zanin E., Wurzenberger C., Braun M., Rabut G., Sironi L., Ellenberg J. (2008). Systematic kinetic analysis of mitotic dis- and reassembly of the nuclear pore in living cells. J. Cell Biol..

[24] Doucet C.M., Talamas J.A., Hetzer M.W. (2010). Cell cycle-dependent differences in nuclear pore complex assembly in metazoa. Cell.

[25] Funakoshi T., Clever M., Watanabe A., Imamoto N. (2011). Localization of Pom121 to the inner nuclear membrane is required for an early step of interphase nuclear pore complex assembly. Mol. Biol. Cell.

[26] Beck M., Hurt E. (2017). The nuclear pore complex: understanding its function through structural insight. Nat. Rev. Mol. Cell Biol..

[27] Walther T.C., Fornerod M., Pickersgill H., Goldberg M., Allen T.D., Mattaj I.W. (2001). The nucleoporin Nup153 is required for nuclear pore basket formation, nuclear pore complex anchoring and import of a subset of nuclear proteins. EMBO J..

[28] D'Angelo M.A., Hetzer M.W. (2008). Structure, dynamics and function of nuclear pore complexes. Trends Cell Biol..

[29] Moudry P., Lukas C., Macurek L., Neumann B., Heriche J.K., Pepperkok R., Ellenberg J., Hodny Z., Lukas J., Bartek J. (2012). Nucleoporin NUP153 guards genome integrity by promoting nuclear import of 53BP1. Cell Death Differ..

[30] Pascon R.C., Miller B.L. (2000). Morphogenesis in Aspergillus nidulans requires Dopey (DopA), a member of a novel family of leucine zipper-like proteins conserved from yeast to humans. Mol. Microbiol..

[31] Mahajan D., Tie H.C., Chen B., Lu L. (2019). Dopey1-Mon2 complex binds to dual-lipids and recruits kinesin-1 for membrane trafficking. Nat. Commun..

[32] Heintzmann R., Huser T. (2017). Super-Resolution Structured Illumination Microscopy. Chem. Rev..

[33] Sobreira N., Schiettecatte F., Valle D., Hamosh A. (2015). GeneMatcher: a matching tool for connecting investigators with an interest in the same gene. Hum. Mutat..

[34] Richards S., Aziz N., Bale S., Bick D., Das S., Gastier-Foster J., Grody W.W., Hegde M., Lyon E., Spector E. (2015). Standards and guidelines for the interpretation of sequence variants: a joint consensus recommendation of the American College of Medical Genetics and Genomics and the Association for Molecular Pathology. Genet. Med..

[35] Winter J.N., Fox T.E., Kester M., Jefferson L.S., Kimball S.R. (2010). Phosphatidic acid mediates activation of mTORC1 through the ERK signaling pathway. Am. J. Physiol. Cell Physiol..

[36] Foster D.A. (2013). Phosphatidic acid and lipid-sensing by mTOR. Trends Endocrinol. Metab..

[37] Nguyen T.B., Louie S.M., Daniele J.R., Tran Q., Dillin A., Zoncu R., Nomura D.K., Olzmann J.A. (2017). DGAT1-Dependent Lipid Droplet Biogenesis Protects Mitochondrial Function during Starvation-Induced Autophagy. Dev. Cell.

[38] LaJoie D., Turkmen A.M., Mackay D.R., Jensen C.C., Aksenova V., Niwa M. (2022). A role for Nup153 in nuclear assembly reveals differential requirements for targeting of nuclear envelope constituents. Mol. Biol. Cell..

[39] Crino P.B. (2011). mTOR: A pathogenic signaling pathway in developmental brain malformations. Trends Mol. Med..

[40] Lipton J.O., Sahin M. (2014). The neurology of mTOR. Neuron.

[41] Bockaert J., Marin P. (2015). mTOR in Brain Physiology and Pathologies. Physiol. Rev..

[42] Fidler D.J., Bailey J.N., Smalley S.L. (2000). Macrocephaly in autism and other pervasive developmental disorders. Dev. Med. Child Neurol..

[43] Klein S., Sharifi-Hannauer P., Martinez-Agosto J.A. (2013). Macrocephaly as a clinical indicator of genetic subtypes in autism. Autism Res..

[44] Webb S.J., Nalty T., Munson J., Brock C., Abbott R., Dawson G. (2007). Rate of head circumference growth as a function of autism diagnosis and history of autistic regression. J. Child Neurol..

[45] Inui M., Miyado M., Igarashi M., Tamano M., Kubo A., Yamashita S., Asahara H., Fukami M., Takada S. (2014). Rapid generation of mouse models with defined point mutations by the CRISPR/Cas9 system. Sci. Rep..

[46] Fujihara Y., Noda T., Kobayashi K., Oji A., Kobayashi S., Matsumura T., Larasati T., Oura S., Kojima-Kita K., Yu Z. (2019). Identification of multiple male reproductive tract-specific proteins that regulate sperm migration through the oviduct in mice. Proc. Natl. Acad. Sci. USA.

[47] Sekiguchi F., Tsurusaki Y., Okamoto N., Teik K.W., Mizuno S., Suzumura H., Isidor B., Ong W.P., Haniffa M., White S.M. (2019). Genetic abnormalities in a large cohort of Coffin-Siris syndrome patients. J. Hum. Genet..

[48] Li H., Durbin R. (2009). Fast and accurate short read alignment with Burrows-Wheeler transform. Bioinformatics.

[49] Peter V.G., Kaminska K., Santos C., Quinodoz M., Cancellieri F., Cisarova K., Pescini Gobert R., Rodrigues R., Custódio S., Paris L.P. (2023). The first genetic landscape of inherited retinal dystrophies in Portuguese patients identifies recurrent homozygous mutations as a frequent cause of pathogenesis. PNAS Nexus.

[50] Wang K., Li M., Hakonarson H. (2010). ANNOVAR: functional annotation of genetic variants from high-throughput sequencing data. Nucleic Acids Res..

[51] Quinodoz M., Peter V.G., Bedoni N., Royer Bertrand B., Cisarova K., Salmaninejad A., Sepahi N., Rodrigues R., Piran M., Mojarrad M. (2021). AutoMap is a high performance homozygosity mapping tool using next-generation sequencing data. Nat. Commun..

[52] Redin C., Gérard B., Lauer J., Herenger Y., Muller J., Quartier A., Masurel-Paulet A., Willems M., Lesca G., El-Chehadeh S. (2014). Efficient strategy for the molecular diagnosis of intellectual disability using targeted high-throughput sequencing. J. Med. Genet..

[53] Nishino K., Toyoda M., Yamazaki-Inoue M., Fukawatase Y., Chikazawa E., Sakaguchi H., Akutsu H., Umezawa A. (2011). DNA methylation dynamics in human induced pluripotent stem cells over time. PLoS Genet..

[54] Hao Y., Hao S., Andersen-Nissen E., Mauck W.M., Zheng S., Butler A., Lee M.J., Wilk A.J., Darby C., Zager M. (2021). Integrated analysis of multimodal single-cell data. Cell.

[55] Pang Z., Chong J., Zhou G., de Lima Morais D.A., Chang L., Barrette M., Gauthier C., Jacques P.É., Li S., Xia J. (2021). MetaboAnalyst 5.0: narrowing the gap between raw spectra and functional insights. Nucleic Acids Res..

[56] Jumper J., Evans R., Pritzel A., Green T., Figurnov M., Ronneberger O., Tunyasuvunakool K., Bates R., Žídek A., Potapenko A. (2021). Highly accurate protein structure prediction with AlphaFold. Nature.

[57] Guerois R., Nielsen J.E., Serrano L. (2002). Predicting changes in the stability of proteins and protein complexes: a study of more than 1000 mutations. J. Mol. Biol..

[58] Schymkowitz J., Borg J., Stricher F., Nys R., Rousseau F., Serrano L. (2005). The FoldX web server: an online force field. Nucleic Acids Res..

