# Supplementary material for: The mTOR-Dop1a-Agpat2 axis regulates nuclear phospholipid homeostasis
Source: iScience. 2026 Apr 22;29(6):115860. doi: 10.1016/j.isci.2026.115860 (PMC13186030; doi:10.1016/j.isci.2026.115860)
Supplement: Document S1. Figures S1–S7 [file mmc1.pdf]

iScience, Volume *29*

Supplemental information

The mTOR-Dop1a-Agpat2 axis regulates nuclear phospholipid homeostasis

Hirotaka Ariyama, Atsushi Tsukamura, Satoko Miyatake, Satoko Okado, Itsuki Itabashi, Ami Ogura, Atsunobu Suzuki, Hyuga Kurakawa, Yuki Sakaguchi, Yuhki Nakatake, Ryunosuke Sanada, Ichiro Terakado, Eriko Koshimizu, Takeshi Mizuguchi, Keisuke Hamada, Kazuhiro Ogata, Eiji Nakagawa, Takafumi Sakakibara, Manabu Shirai, Yoshitaka Fujihara, Mukhtar Ullah, Mathieu Quinodoz, Carlo Rivolta, Abdul Ghafoor Khan, Muhammad Nadeem Khan, Muhammad Ansar, Erica H. Gerkes, Tuula Rinne, Alexander P.A. Stegmann, Margje Sinnema, Malak Ali Alghamdi, Essa Alharby, Reham M. Balahmar, Naif A.M. Almontashiri, Sarah Baer, Amélie Piton, Carla Díes Curià, Sandra Mercier, Benjamin Cogné, Patrick Yap, Shin-ya Morita, Akiyoshi Kakita, Mitsuhiro Kato, Yoshihiro Maruo, Naomichi Matsumoto, and Masaki Mori

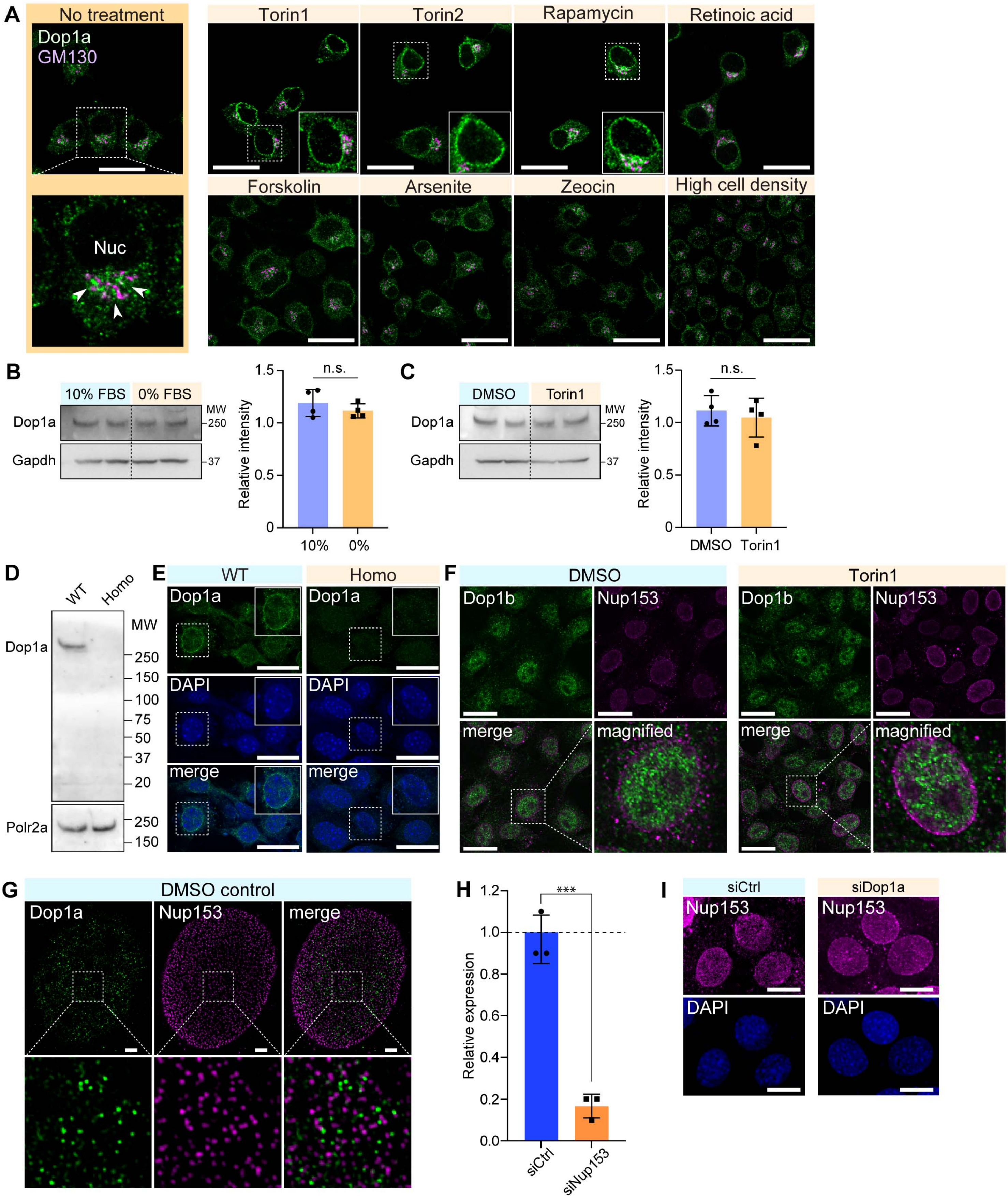
A
No treatment
Dop1a
GM130
Nuc
Torin1
Torin2
Rapamycin
Retinoic acid
Forskolin
Arsenite
Zeocin
High cell density
B
10% FBS
0% FBS
Dop1a
Gapdh
MW
250
37
Relative intensity
n.s.
10%
0%
C
DMSO
Torin1
Dop1a
Gapdh
n.s.
D
WT
Homo
Dop1a
Polr2a
MW
250
150
100
75
50
37
20
E
WT
Homo
Dop1a
DAPI
merge
F
DMSO
Torin1
Dop1b
Nup153
merge
magnified
G
DMSO control
Dop1a
Nup153
merge
H
Relative expression
***
siCtrl
siNup153
I
siCtrl
siDop1a
Nup153
DAPI

Figure S1. Expression analysis of Dop1a. Related to Figure 1.

(A) Immunostaining of Dop1a in Neuro2a cells treated with compounds for 24 hrs. GM130 antibody was used to stain the Golgi apparatus. Arrowheads indicate Dop1a signals colocalising with GM130. Scale bar = 20 μm. Nuc, nucleus.

(B) Immunoblot analysis of Dop1a in Neuro2a cells cultured in 10% FBS or 0% FBS for 16 h. Gapdh was used as a loading control. The right panel shows densitometry of Dop1a normalized to Gapdh (n = 4). (C) Immunoblot analysis of Dop1a in Neuro2a cells treated with DMSO or Torin1 for 16 h. Gapdh served as a loading control. The right panel shows densitometry of Dop1a normalized to Gapdh (n = 4). (D) Western blot for Dop1a in Dop1a-KO MEFs. Polr2a was used as a loading control. (E) Immunofluorescence of Dop1a in Dop1a-KO MEFs treated with Torin1. No appreciable signal was observed by the Dop1a staining in Dop1a-KO MEFs. Nuclei were stained with DAPI. Scale bar = 20 μm.

(F) Immunostaining of Dop1b/Dopey2, showing localization to nucleoplasm in cells treated with Torin1 or DMSO. The NM was stained with the Nup153 antibody. Scale bar = 20 μm. (G) SIM imaging of Dop1a and Nup153 in Neuro2a cells under control conditions. Neuro2a cells were immunostained for Dop1a (green) and Nup153 (magenta) and imaged using super-resolution SIM microscopy in the absence of Torin1 (DMSO control). The dashed boxes indicate regions that are shown at higher magnification below. Dop1a and Nup153 showed non-overlapping distributions, with a Pearson' s correlation coefficient (PCC) = −0.02. Scale bar, 1 μm. (H) Real-time qPCR for *Nup153* in cells transfected with control siRNA or siRNA against *Nup153* (n = 3). *Tubb5* was used to normalize the expression levels. ***$p < 0.001$, unpaired two-tailed Student' s *t*-test. (I) Immunofluorescence analysis of Nup153 in Neuro2a cells following *Dop1a* knockdown. Neuro2a cells were transfected with siCtrl or siDop1a. After 48 h, cells were fixed and immunostained with an antibody against Nup153 (magenta) and counterstained with DAPI (blue). Scale bar, 10 μm.

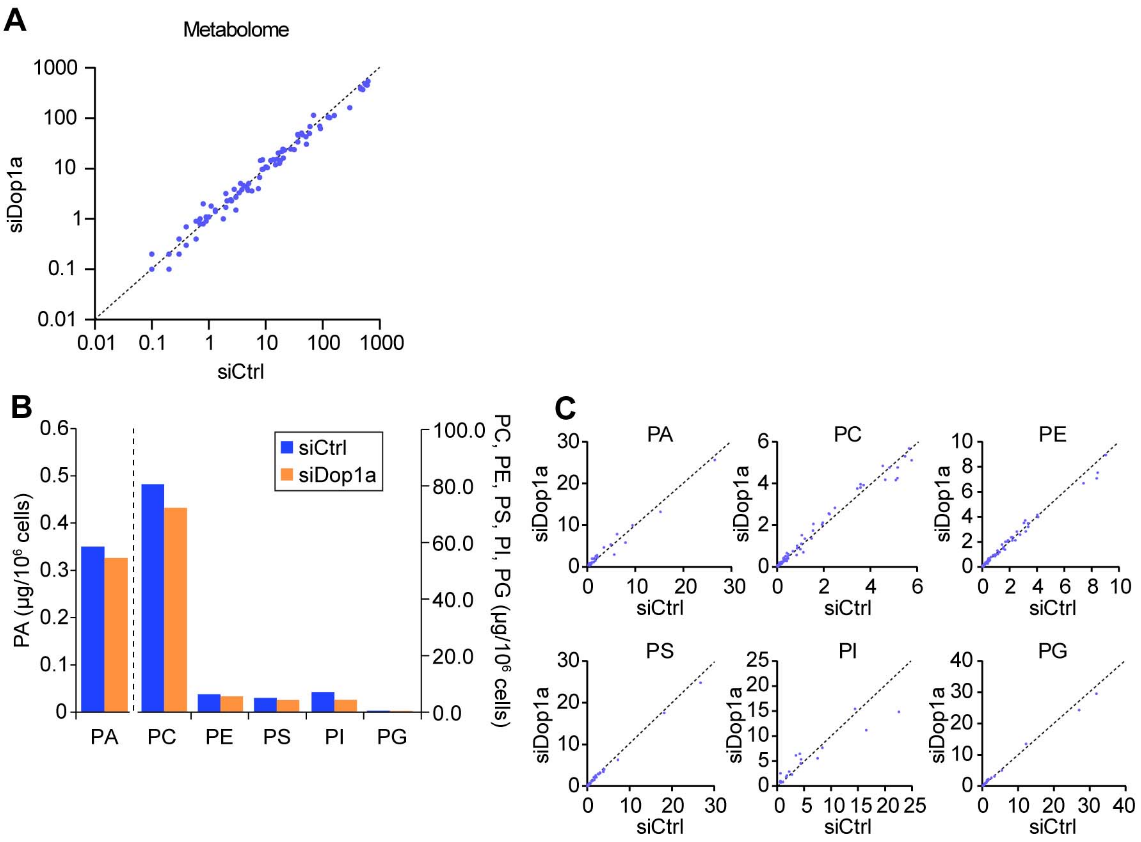

Figure S2. Metabolome analyses. Related to Figure 2.
(A) Comprehensive metabolome profiling of siCtrl- and siDop1a-transfected Neuro2a cells under the normal 10% FBS condition. Scatter plot showing relative metabolite abundances in siDop1a- versus siCtrl-treated cells. Signal intensity was expressed as peak area normalized to the common internal standards, displayed on log-transformed axes.
(B) Quantification of PA, PC, PE, PS, PI, and PG in Neuro2a cells following siCtrl or siDop1a transfection, without Torin1 treatment. PA is plotted on the left axis (μg/10^6 cells), and PC, PE, PS, PI, and PG are plotted on the right axis (μg/10^6 cells). (C) Scatter plots comparing the concentrations of individual molecular species for PA, PC, PE, PS, PI, and PG in siDop1a- versus siCtrl-transfected Neuro2a cells (% of total PLs). Dashed lines indicate the line of identity.

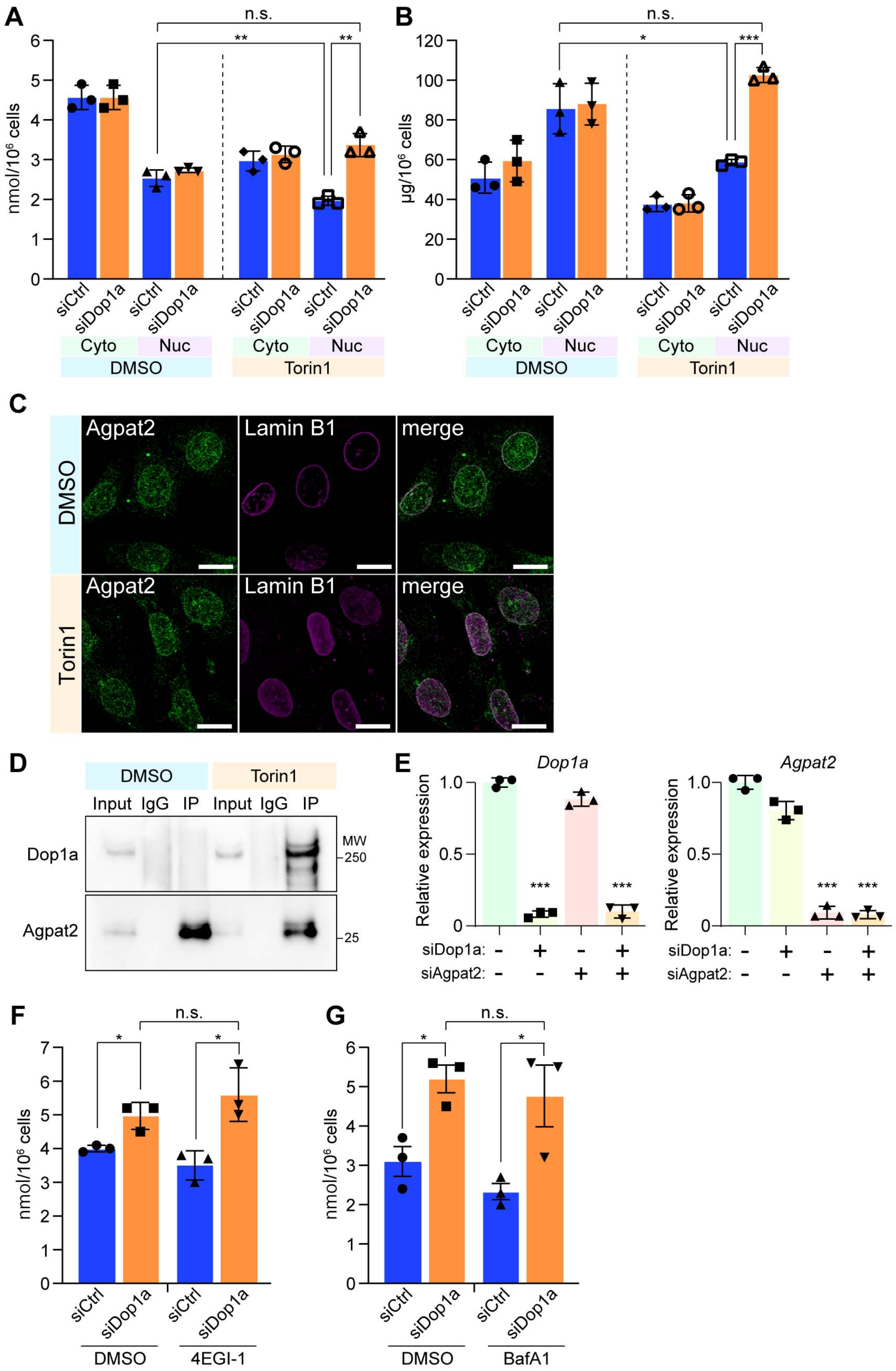
A
n.s.
**
**
nmol/10⁶ cells
siCtrl
siDop1a
Cyto
Nuc
DMSO
Torin1
B
n.s.
*
***
μg/10⁶ cells
C
Agpat2
Lamin B1
merge
DMSO
Torin1
D
DMSO
Torin1
Input
IgG
IP
Dop1a
Agpat2
MW
250
25
E
Dop1a
Agpat2
Relative expression
***
siDop1a:
siAgpat2:
F
n.s.
*
DMSO
4EGI-1
G
n.s.
*
BafA1

Figure S3. Mechanistic analysis of PL metabolism. Related to Figure 3.
(A) PA content in cytosolic (Cyto) and nuclear (Nuc) fractions of Neuro2a cells transfected with siCtrl or siDop1a and treated with Torin1 or DMSO (48 h). Bars indicate mean ± s.d. from n = 3 biological replicates. (B) PC content in the Cyto and Nuc fractions of Neuro2a cells transfected with siCtrl or siDop1a and treated with Torin1 or DMSO (48 h). Bars indicate mean ± s.d. from n = 3 biological replicates. *$p < 0.05$, **$p < 0.01$, and ***$p < 0.001$, unpaired two-tailed Student’ s *t*-test. (C) Immunostaining of Agpat2 in NIH3T3 cells. The NM was stained with a Lamin B1 antibody, as shown in magenta. Scale bar = 10 µm. (D) Co-immunoprecipitation using an Agpat2 antibody in Neuro2a cells treated with Torin1 or DMSO. The immunoblot shows proteins co-precipitating with Agpat2. (E) Knockdown efficiency of *Dop1a* and *Agpat2* in the Neuro2a cells used for PL-MS analysis. Data were normalized to *Gapdh* expression. Data are represented as means ± SEM (n = 3). ***$p < 0.001$, unpaired two-tailed Student’ s *t*-test. (F) PA assay was performed on Neuro2a cells transfected with siCtrl or siDop1a and subsequently treated for 48 h with 4EGI-1 or DMSO in the presence of Torin1 (n = 3 biological replicates). Bars show mean ± s.d. *$p < 0.05$; n.s., not significant. (G) PA assay was performed on Neuro2a cells transfected with siCtrl or siDop1a and treated for 48 h with bafilomycin A1 (BafA1) or DMSO in the presence of Torin1 (n = 3 biological replicates). Bars show mean ± s.d. *$p < 0.05$; n.s., not significant.

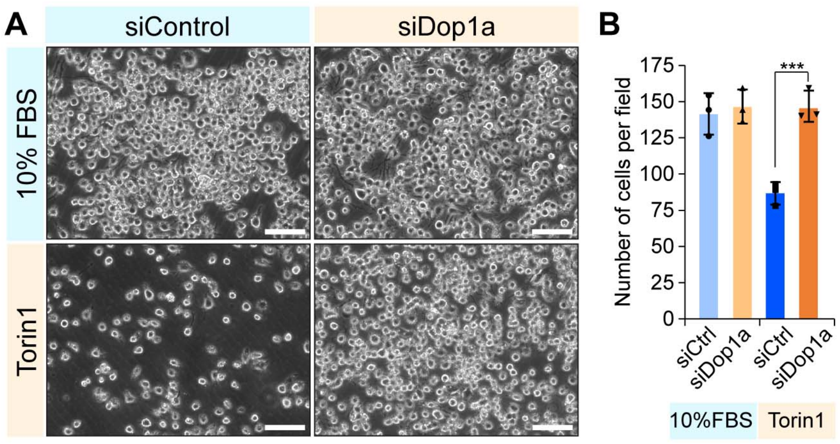

Figure S4. Cell cycle analysis of Dop1a-depleted cells. Related to Figure 4.
(A) Phase-contrast images of Neuro2a cells depleted of *Dop1a* or left non-depleted, followed by serum deprivation. Scale bar = 50 µm. (B) Quantification of cell numbers under serum-starved conditions in *Dop1a*-depleted Neuro2a cells. Data are presented as mean ± SEM (n = 3 biological replicates). ***$p < 0.001$, unpaired two-tailed Student' s *t*-test.

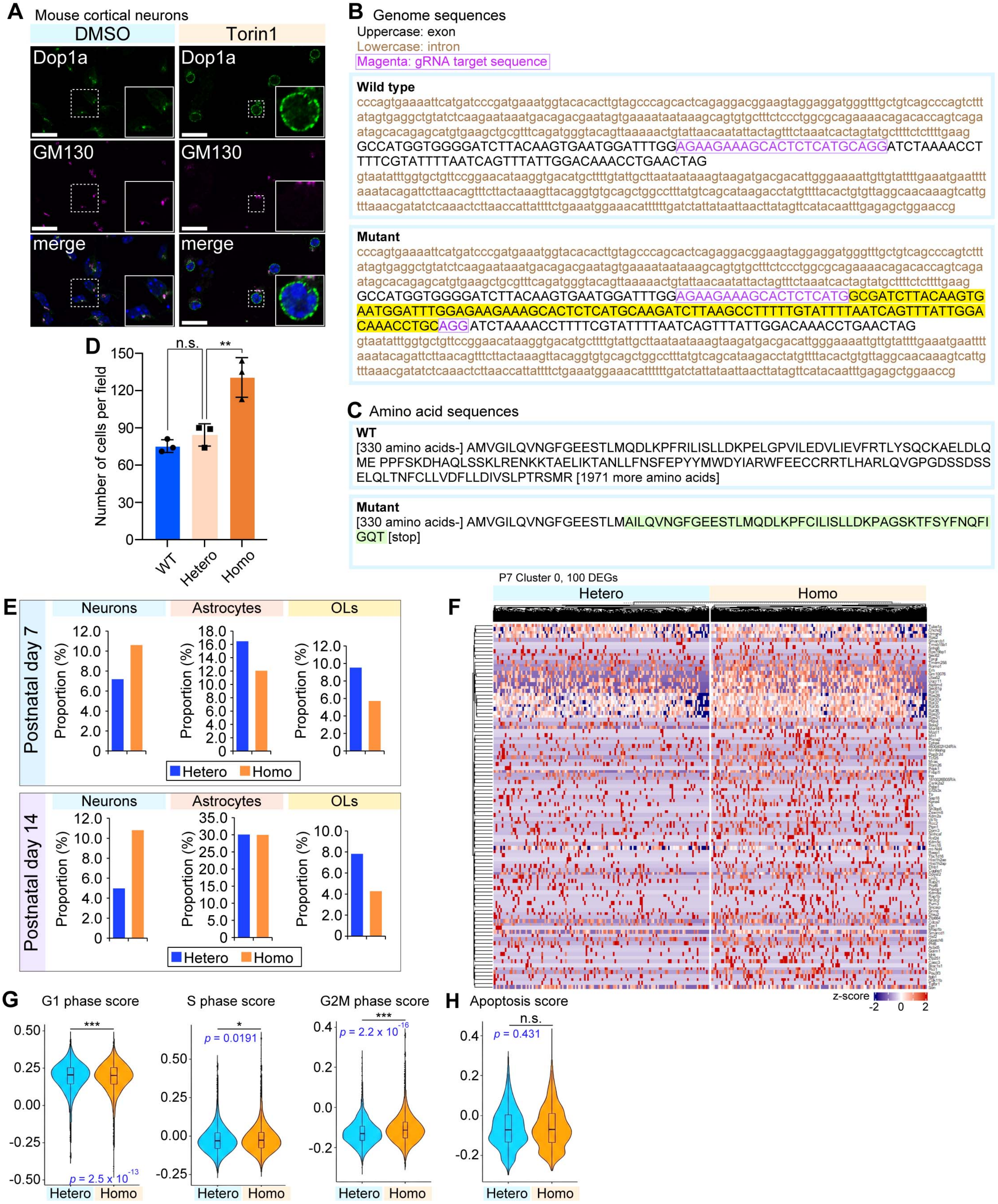
A
Mouse cortical neurons
DMSO
Torin1
Dop1a
GM130
merge
B
Genome sequences
Uppercase: exon
Lowercase: intron
Magenta: gRNA target sequence
Wild type
cccagtgaaaattcatgatcccgatgaaatggtacacacttgtagcccagcactcagaggacggaagtaggaggatgggtttgctgtcagcccagtcttt
atagtgaggctgtatctcaagaataaatgacagacgaatagtgaaaataataaagcagtgtgctttctccctggcgcagaaaacagacaccagtcaga
atagcacagagcatgtgaagctgcgtttcagatgggtacagttaaaaactgtattaacaatattactagtttctaaatcactagtatgcttttctcttttgaag
GCCATGGTGGGGATCTTACAAGTGAATGGATTTGGAGAAGAAAGCACTCTCATGCAGGATCTAAAACCT
TTTCGTATTTTAATCAGTTTATTGGACAAACCTGAACTAG
gtaatatttggtgctgttccggaacataaggtgacatgcttttgtattgcttaataataaagtaagatgacgacattgggaaaattgttgtattttgaaatgaatttt
aaatacagattcttaacagtttcttactaaagttacaggtgtgcagctggcccttttatgtcagcataagacctatgttttacactgtgttaggcaacaaagtcattg
tttaaacgatatctcaaactcttaaccattattttctgaaatggaaacatttttgatctattataattaacttatagttcatacaatttgagagctggaaccg
Mutant
cccagtgaaaattcatgatcccgatgaaatggtacacacttgtagcccagcactcagaggacggaagtaggaggatgggtttgctgtcagcccagtcttt
atagtgaggctgtatctcaagaataaatgacagacgaatagtgaaaataataaagcagtgtgctttctccctggcgcagaaaacagacaccagtcaga
atagcacagagcatgtgaagctgcgtttcagatgggtacagttaaaaactgtattaacaatattactagtttctaaatcactagtatgcttttctcttttgaag
GCCATGGTGGGGATCTTACAAGTGAATGGATTTGGAGAAGAAAGCACTCTCATGGCGATCTTACAAGTG
AATGGATTTGGAGAAGAAAGCACTCTCATGCAAGATCTTAAGCCTTTTTGTATTTTAATCAGTTTATTGGA
CAAACCTGCAGGATCTAAAACCTTTTCGTATTTTAATCAGTTTATTGGACAAACCTGAACTAG
gtaatatttggtgctgttccggaacataaggtgacatgcttttgtattgcttaataataaagtaagatgacgacattgggaaaattgttgtattttgaaatgaatttt
aaatacagattcttaacagtttcttactaaagttacaggtgtgcagctggcccttttatgtcagcataagacctatgttttacactgtgttaggcaacaaagtcattg
tttaaacgatatctcaaactcttaaccattattttctgaaatggaaacatttttgatctattataattaacttatagttcatacaatttgagagctggaaccg
C
Amino acid sequences
WT
[330 amino acids-] AMVGILQVNGFGEESTLMQDLKPFRILISLLDKPELGPVILEDVLIEVFRTLYSQCKAELDLQ
ME PPFSKDHAQLSSKLRENKKTAELIKTANLLFNSFEPYYMWDYIARWFEECCRRTLHARLQVGPGDSSDSS
ELQLTNFCLLVDFLLDIVSLPTRSMR [1971 more amino acids]
Mutant
[330 amino acids-] AMVGILQVNGFGEESTLMAILQVNGFGEESTLMQDLKPFCILISLLDKPAGSKTFSYFNQFI
GQT [stop]
D
n.s.
**
Number of cells per field
150
120
90
60
30
0
WT
Hetero
Homo
E
Postnatal day 7
Postnatal day 14
Neurons
Astrocytes
OLs
Proportion (%)
Hetero
Homo
F
P7 Cluster 0, 100 DEGs
Hetero
Homo
z-score
-2
0
2
G
G1 phase score
***
p = 2.5 x 10^-13
S phase score
*
p = 0.0191
G2M phase score
***
p = 2.2 x 10^-16
Hetero
Homo
H
Apoptosis score
n.s.
p = 0.431
Hetero
Homo

Figure S5. Generation of the Dop1a KO mouse strains. Related to Figure 5.
(A) Immunofluorescence of Dop1a in primary neurons isolated from neonatal mouse cerebral cortex. Nuclei were stained with DAPI. Scale bar = 20 µm. (B) The genome sequences of Dop1a mutant strains in which the highlighted sequence was inserted, causing the frameshift mutation. (C) Amino acid sequence predicted to be expressed by the +94 mutant. Highlighted is the amino acid sequence generated by the frameshift and terminated prematurely. (D) Number of cells per field in WT, heterozygous *Dop1a* KO (Hetero), and homozygous *Dop1a* KO (Homo) MEFs 72 h after Torin1 treatment. Data represent mean ± SEM. Statistical significance was assessed by one-way ANOVA followed by Tukey' s post-hoc test; n.s., not significant; $^{**}p < 0.01$. (E) Cluster sizes of neurons, astrocytes, and OLs. (F) Heatmap of the top 100 differentially expressed genes in Cluster 0 at P7, presented as z-score–normalized expression values to illustrate transcriptional alterations caused by Dop1a homozygous mutation. (G) Violin plots of G1, S, and G2/M cell-cycle phase scores derived from scRNA-seq profiles in Cluster 0. Homozygous *Dop1a* mutant cells show reduced G1-phase scores and elevated S and G2/M scores relative to heterozygous samples ($p = 2.5 \times 10^{-13}$ for G1; $p = 0.0191$ for S; $p = 2.2 \times 10^{-16}$ for G2/M). (H) Apoptosis module scores in Cluster 0 cells at P7, showing no significant difference between genotypes (p = 0.431).

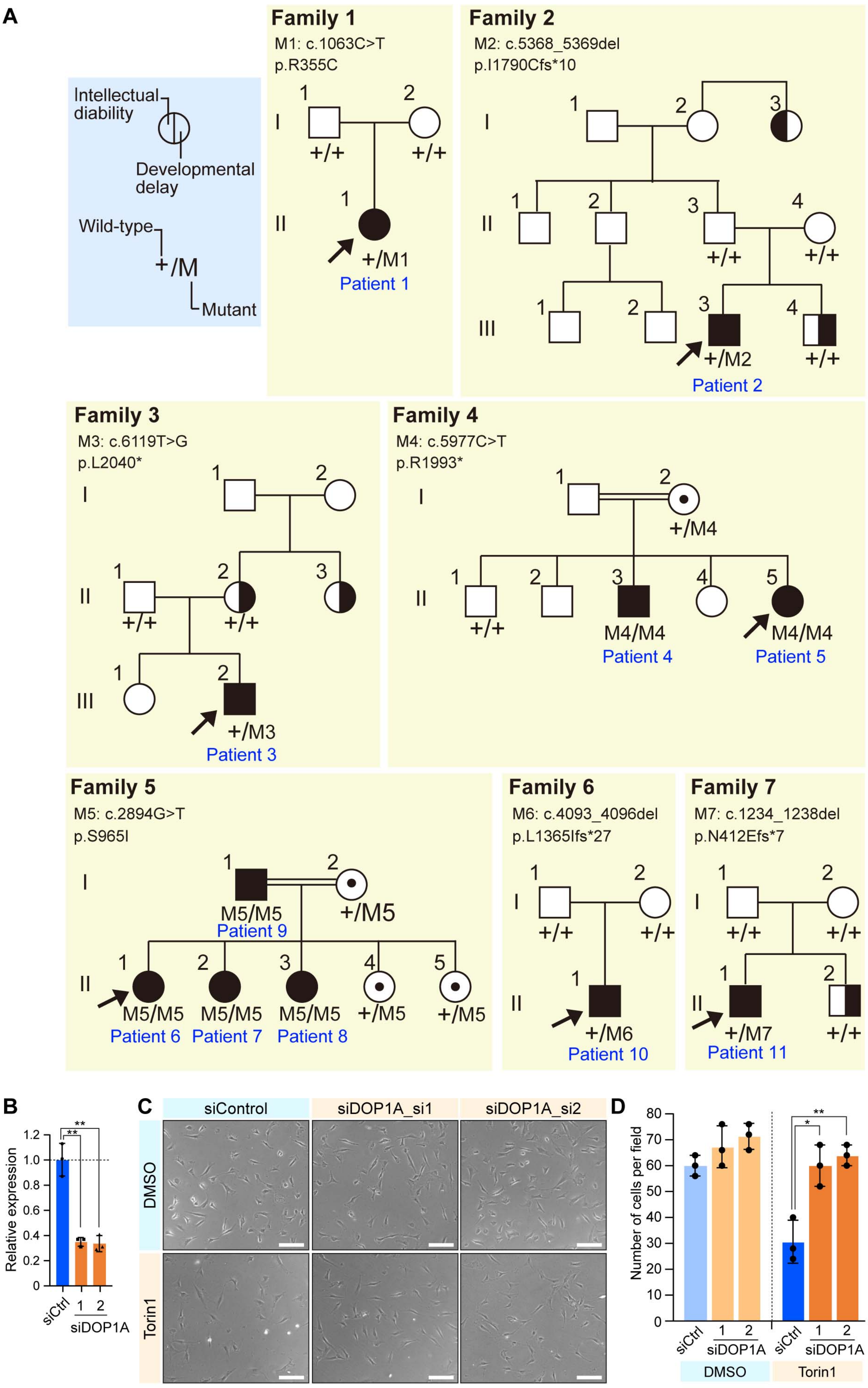

A
Intellectual diability
Developmental delay
Wild-type
+/M
Mutant
Family 1
M1: c.1063C>T
p.R355C
+/+
+/M1
Patient 1
Family 2
M2: c.5368_5369del
p.I1790Cfs*10
+/M2
Patient 2
Family 3
M3: c.6119T>G
p.L2040*
+/M3
Patient 3
Family 4
M4: c.5977C>T
p.R1993*
+/M4
M4/M4
Patient 4
Patient 5
Family 5
M5: c.2894G>T
p.S965I
M5/M5
+/M5
Patient 9
Patient 6
Patient 7
Patient 8
Family 6
M6: c.4093_4096del
p.L1365Ifs*27
+/M6
Patient 10
Family 7
M7: c.1234_1238del
p.N412Efs*7
+/M7
Patient 11
B
Relative expression
siCtrl
siDOP1A
C
siControl
siDOP1A_si1
siDOP1A_si2
DMSO
Torin1
D
Number of cells per field
siCtrl
siDOP1A
DMSO
Torin1

Figure S6. DOP1A variants identified in NDD patients and functional analysis in human iPSC-derived neural cells. Related to Figure 6.
(A) Black squares and black circles indicate affected male and female individuals, respectively. Arrows indicate probands. Individual symbols are partitioned, indicating the presence of intellectual disability (left) and developmental delay (right), respectively. (B) Knockdown efficiency of *DOP1A*. Human induced pluripotent stem cells (hiPSCs) were differentiated into NSCs and transfected with siCtrl or two different siRNAs targeting *DOP1A* (siDOP1A_si1 or siDOP1A_si2). *DOP1A* mRNA levels were quantified 72 h after transfection and normalized to *GAPDH* (n = 3 biological replicates). (C) Representative phase-contrast images of hiPSC-derived NSCs. After siRNA transfection, cells were treated with DMSO or Torin1 and cultured for 48 h for imaging. Scale bar, 100 µm. (D) Quantification of cell number per field. Cells were counted across biological replicates (n = 3). Bars represent mean ± SD. Statistical significance was determined by one-way ANOVA with post hoc testing (*$p < 0.05$; **$p < 0.01$).

A Genome sequences of Dop1a WT and R355C allele

Uppercase: exon
Lowercase: intron
Magenta: gRNA target sequence

Wild type allele

cccagtgaaaattcatgatcccgatgaaatggtacacacttgtagcccagcactcagaggacggaagtaggaggatgggtttgctgtcagcccagtcttt
atagtgaggctgtatctcaagaataaatgacagacgaatagtgaaaataataaagcagtgtgctttctccctggcgcagaaaacagacaccagtcaga
atagcacagagcatgtgaagctgcgtttcagatgggtacagttaaaaactgtattaacaatattactagtttctaaatcactagtatgcttttctcttttgaag
GCCATGGTGGGGATCTTACAAGTGAATGGATTTGGAGAAGAAAGCACTCTCATGCAGGATCTAAAACCT
TTTCGTATTTTAATCAGTTTATTGGACAAACCTGAACTAG
gtaatatttggtgctgttccggaacataaggtgacatgcttttgtattgcttaataataaagtaagatgacgacattgggaaaattgttgtattttgaaatgaatttt
aaatacagattcttaacagtttcttactaaagttacaggtgtgcagctggcctttatgtcagcataagacctatgttttacactgtgttaggcaacaaagtcattg
tttaaacgatatctcaaactcttaaccattattttctgaaatggaaacattttttgatctattataattaacttatagttcatacaatttgagagctggaaccg

R355C mutant allele

, substituted base
, AseI site

cccagtgaaaattcatgatcccgatgaaatggtacacacttgtagcccagcactcagaggacggaagtaggaggatgggtttgctgtcagcccagtcttt
atagtgaggctgtatctcaagaataaatgacagacgaatagtgaaaataataaagcagtgtgctttctccctggcgcagaaaacagacaccagtcaga
atagcacagagcatgtgaagctgcgtttcagatgggtacagttaaaaactgtattaacaatattactagtttctaaatcactagtatgcttttctcttttgaag
GCCATGGTGGGGATCTTACAAGTGAATGGATTTGGAGAAGAAAGCACTCTCATGCAGGATCTAAAACCT
TTTTGTATATTAATCAGTTTATTGGACAAACCTGAACTAG
gtaatatttggtgctgttccggaacataaggtgacatgcttttgtattgcttaataataaagtaagatgacgacattgggaaaattgttgtattttgaaatgaatttt
aaatacagattcttaacagtttcttactaaagttacaggtgtgcagctggcctttatgtcagcataagacctatgttttacactgtgttaggcaacaaagtcattg
tttaaacgatatctcaaactcttaaccattattttctgaaatggaaacattttttgatctattataattaacttatagttcatacaatttgagagctggaaccg

B

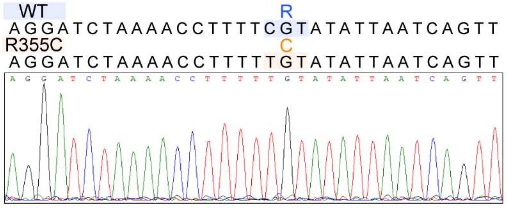

Figure S7. Generation of the Dop1a R355C mouse strains. Related to Figure 7.
(A) The genome sequence of the Dop1a R355C allele. The base substitution from C to T is highlighted to introduce the R355C amino acid substitution. The boxed sequence is the AseI restriction site introduced via a silent mutation for the convenience of genotyping. (B) The chromatogram of the Dop1a-R355C homozygous mouse.

Data S1: Uncropped western blot images

Figure 1F

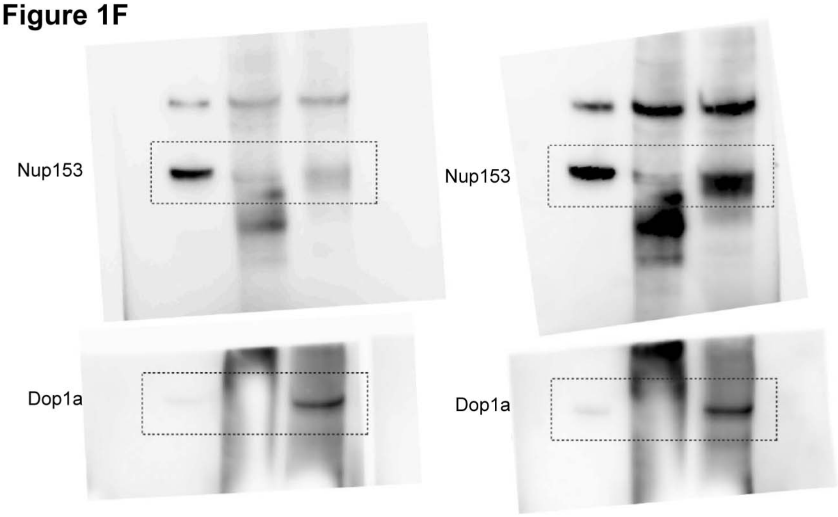

Figure 3A

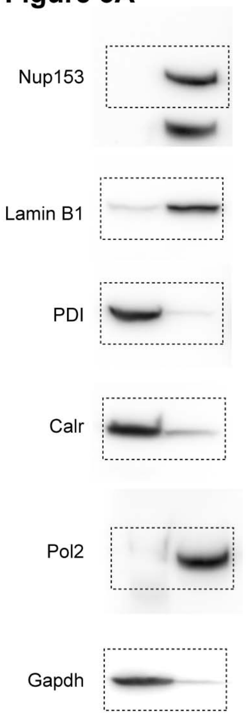

Figure 2A

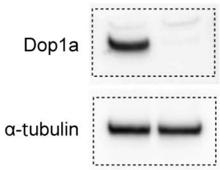

Figure 5C

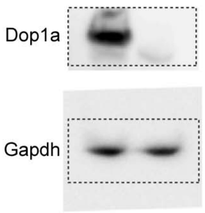

Figure S1B

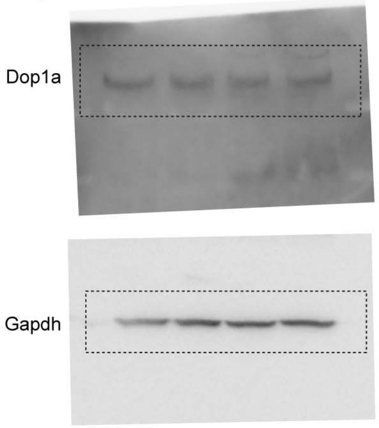

Figure S1C

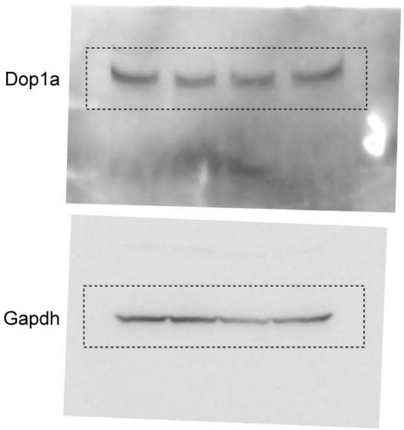

Figure S1D

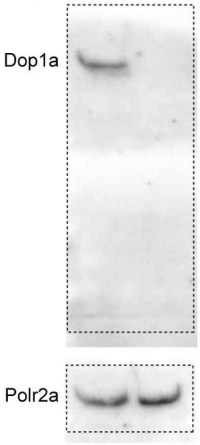

Figure S3D

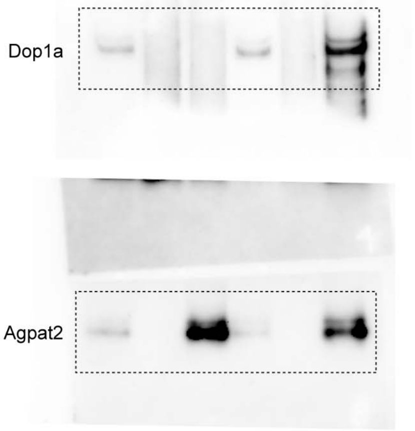

Clinical summaries of patients with *DOP1A* variants

Patient 1 in Family 1

This is a 14-year-old girl born to non-consanguineous Japanese parents. She was born at 40 weeks and one day of gestation by spontaneous delivery without any complications after an uneventful pregnancy. Her birth weight was 2880 g (-0.3 SD), and her height was 50 cm (+0.8 SD). At the age of three days, a tonic spasm consisting of a "hiccup-like" jerk of the trunk and bilateral upper and lower extremities and blepharospasm first appeared and lasted about 20 seconds. Tonic spasms were increasingly observed on the fifth and ninth day after birth. At the age of 10 days, tonic spasms happened in clusters, and she was admitted to the emergency hospital. After starting phenobarbital therapy, the spasms disappeared for a week, but they appeared again at the age of 18 days. There were 10-15 spasms in a cluster, and up to 30 clusters were observed daily, irrespective of whether they were awake or asleep. Vitamin B6 and zonisamide were not effective.

Laboratory blood and urine tests (blood count, biochemical exam including serum magnesium, vitamin B6, lactic acid, pyruvate, metabolic analyses of blood and urine, TORCH infections, chromosomal G-banding), cerebrospinal fluid analysis including lactate and pyruvate, fundus examination, chest X-ray, and echocardiography were performed, but no abnormalities were noted.

Electroencephalogram (EEG) showed a suppression-burst pattern with left occipital dominance. Brain MRI showed left hemimegaloencephaly without intracerebral calcification or intraventricular nodules. She was diagnosed with early infantile epileptic encephalopathy with suppression burst (EIEE), possibly due to left hemimegaloencephaly. Tuberous sclerosis or neurocutaneous syndromes were unlikely without any skin lesions. Since increased tonic spasms and status epilepticus were observed and antiepileptic drugs were ineffective, left hemispherectomy was performed at the age of 2 months. After the surgery, the spasms did not worsen, and she was discharged from the hospital. At three years and eight months, tonic spasms lasting 1-2 seconds, occurring in clusters, were observed daily. EEG was performed, showing active multifocal spikes without hypsarrhythmia.

Her height and weight were small for her age (-1.31 SD and -0.3 SD, respectively), and her head circumference was large for her age (+1.69 SD). She had a severe DD with the achieved developmental milestones of head control, sitting with support, eye fixation, following objects, and social smiling at that time. She showed hypotonia with frog leg posture and hyperreflexia. No involuntary movement was observed. She did not achieve any further psychomotor development at seven years and six months. She had clustered tonic spasms

daily, presented as the most severe form of epileptic encephalopathy. By age 10, she had gradually caught up in her development. She could go up and down stairs using handrails and eat alone. Her IQ was unknown. She attended a special support class at elementary school. She has tonic seizures every day. Trio-based exome sequencing was performed, identifying a *de novo* missense variant in the *DOP1A* [NM_015018.3: c.1063C>T p.(R355C)].

Patient 2 in Family 2

This is a 17-year-old boy born to non-consanguineous Caucasian parents. During pregnancy, the mother was diagnosed with hypothyroidism, for which substitution therapy was needed. The pregnancy was otherwise uncomplicated. He was born at 42 weeks of gestation after a long parturition, followed by an emergency cesarean section. He showed a good Apgar score with a birth weight of 4385 g. He underwent phototherapy in connection with hyperbilirubinemia, and he was discharged from the hospital at the age of seven days. No hypotonia or feeding problems were observed in his neonatal period. He achieved delayed motor milestones, such as walking independently by his 2nd year, and delayed but relatively smooth speech development, as well as singing songs by the age of 2.5. At a young age, he could amuse himself for hours with a toy in the playpen. At preschool, he was often noticed screaming and panicking quickly. He soon moved to a specialized daycare and took a special education class. Recently, he entered an institution for people with IDs. He has been diagnosed with autism spectrum disorder (ASD) with very mild ID (IQ: 61-71). He exhibited an inharmonious profile, with a social and emotional level comparable to that of 0-3 years. He had been admitted to the hospital once because of a substantial amount of pericardial fluid, for which no apparent cause was found, and no recurrence was observed.

As for the family history, He has a brother who also showed DD at an early age and has been diagnosed with ASD with borderline intelligence (IQ: 70) and a social-emotional level of 3-7 years. His brother is generally more mildly affected than the index patient. His grandmother was diagnosed with ASD, and her sister had ASD with ID.

Exome sequencing identified a *de novo* frame-shift variant in the *DOP1A* [NM_015018.3: c.5368_5369del p.(I1790Cfs*10)], only in the proband, but no variant in the affected brother.

Patient 3 in Family 3

This is a 13-year-old boy born to non-consanguineous Caucasian parents. The age at the last visit was four years and eight months. He was born by a cesarean section because of abnormal positioning after an uneventful pregnancy. Birth weight was within normal limits [3,990 kgs at 39+6 weeks, (+0.63 SD)]. He had a good start. After that, he was hypotonic and had feeding problems and reflux, for which he was admitted to the neonatal ward for

observation twice in the first two months. He has a global DD. He started walking at 24.5 months. He began speaking two-word sentences around the age of three. At the age of 4, his motor development was delayed globally but progressed slowly. He had tonal dysregulation. At the age of five years, he developed type 1 diabetes mellitus. He did not show overt neurological problems at age 5, apart from motor delay and clumsiness. At age five, he was diagnosed with autism. He attended special education. IQ at age three years was 76. He underwent routine biometry at the ages of four and eight months, revealing a flat philtrum and a broad nasal bridge. He had one large hyperpigmentation on his skin (10 x 15 cm) and one cafe-au-lait macule on his right upper leg. IQ at the age of five years and eight months was 60. His mother has complex psychiatric problems for which hospitalization was needed. She attended special education as a child. The sister of the mother had learning problems but no psychiatric problems. His father did not have developmental or learning problems. He has high myopia, like his sisters. He has a sister with typical development. SNP microarray revealed 1,7 Mb paternal duplication 3q13.13 and 216 kb maternal duplication 9p24.1. Both duplications are considered probably benign. The *FMR1* gene testing was negative, with an average CGG repeat count. Metabolic testing showed no cause for the DD. The mtDNA analysis showed no mutation. Exome sequencing in trio analysis performed in 2016 showed a *de novo* nonsense variant in the *DOP1A* [NM_015018.3:c.6119T>G p.(L2040*)].

Patients 4 and 5 in Family 4

The parents were first-degree cousins from the Khyber Pakhtunkhwa region of Pakistan and had five children. Two of them, a male and a female, exhibited severe intellectual DD. Specifically, patient 4 showed severe to profound ID associated with fits, aphasia, challenging behaviors, and stereotypical movements. Patient 5 showed moderate to severe ID, aggressive outbursts, was overweight, and had stereotypical behaviors. A functional examination of the brain was conducted using an EEG in the awake state. The background recording showed regular rhythmical activity, which was mainly alpha-range activity. No abnormal discharges, focal or generalized epileptiform, were recorded in the awake state. Photic stimulation did not evoke any abnormal response and revealed no changes. We could not perform a brain MRI because the parents refused total anesthesia. We also analyzed cardiac function using electrocardiography (ECG) and echocardiography (ECHO). The ECG examination revealed a normal heart rhythm without any pathological signs. Similarly, ECHOs did not reveal any functional or mechanical defects in the hearts of the two patients. The fractional shortening and ejection fraction in patient 4 were 31% and 63%, respectively. His sister (patient 5) exhibited almost the same values, 32% and 65%. The M-mode and 2D

echocardiography revealed no changes in cardiac chamber size, wall motion, or valvular structures. No pericardial effusion and left ventricular/atrial clots were seen in either patient. In conclusion, cardiac assessment revealed preserved left ventricular function in both patients.

Exome sequencing was performed on patient 5. AutoMap-mediated analysis revealed an elevated level of genome-wide homozygosity (392.83 Mb), likely due to the parents being genetically related. However, no convincing biallelic candidate mutations in autozygous regions or elsewhere were identified in genes previously known to be linked to ID, as reported by the OMIM database (McKusick, 2007). We identified a homozygous nonsense variant in *DOP1A* [NM_015018.3: c.5977C>T p.(R1993*)] in both patients. The variant is also in a sizeable homozygous region (25.46 Mb). Homozygosity for this change, assessed by targeted Sanger sequencing, segregated with disease in both patients, whereas their mother was a heterozygous carrier, and their eldest unaffected brother had a WT genotype at this site.

Patients 6-9 in Family 5

In this family, four patients [I-1 (father) and II-1, 2, and 3 (three children)] had a homozygous missense variant in *DOP1A* [NM_015018.3:c.2894G>T, p.(S965I)]. Three individuals [I-2 (mother) and II-4 and II-5 (two other children)] had a heterozygous variant. Five children were born to parents who are consanguineous. None of the individuals with the heterozygous variant shows any notable symptoms. In contrast, all patients with the homozygous variant have a variable range of symptoms, from delayed walking and speech to moderate to severe ID.

For the three affected children (II-1, 2, and 3), their pregnancies were normal except for II-1, who showed intrauterine growth retardation. They were all born at full term. II-2 and 3 had average weight, length, and head circumference at birth, while II-1 was generally small for age.

None of the four patients (I-1, II-1, II-2, and II-3) exhibited dysmorphology; however, II-2 and II-3 failed to thrive in height and weight, reaching the 3rd centile at the ages of 4 years and 3 years, respectively. II-1 showed significant microcephaly with head circumference below the 3rd centile at the age of 7 years, and II-2 also showed a small head size with HC on the 5th centile at four years. II-1 and II-3 showed moderate DD and ID, while I-1 had motor DD and speech and language delay without ID, and II-2 showed mild motor DD, severe speech and language delay, and autistic features without apparent ID (IQ 90). However, severe speech and language delays are observed in all four patients. Ocular features, including nystagmus and strabismus, were observed in II-1 and II-2. In II-1 and II-3, brain MRI detected the congenital hypogenesis or partial agenesis of the corpus callosum.

Additionally, a small white matter lesion, suspected to be a result of perinatal hypoxic insult, was noted in II-1. The homozygous c.2894G>T p.(S965I) *DOP1A* variant was detected by whole-exome sequencing performed on II-1, II-3, and I-1. Sanger sequencing confirmed the genotypes in this family.

Patient 10 in Family 6

Patient 10 is a 10-year-old French boy at the time of the study. No apparent family history was noted, and the pregnancy and birth were uncomplicated. He showed delayed developmental milestones (first words at 2.5 years, walking at three years, first sentences at five years, toilet training at five years) and had a moderate ID. Dysmorphic features of a high forehead, flat nasal root, deep-set eyes, and microretrognathia were observed. He has strabismus. Behavioral problems such as anxiety, frustration intolerance, and anger are currently major issues for this child. Brain MRI revealed atrophy of the corpus callosum, brainstem, and progressive cerebellar atrophy. Clinically, at the age of 6 years old, he presented with strabismus, hand-eye coordination disorder, global hypotonia, and inability to walk on a line. Trio-based whole genome sequencing detected the de novo c.4093_4096del p.(L1365Ifs*27) variant, which was confirmed by Sanger sequencing.

Patient 11 in Family 7

Patient 11 is an 8-year-old French boy at the time of the study. He has joint hyperlaxity and a short uvula as a minor dysmorphological feature. One single café au lait spot was observed on his skin. He showed delayed developmental milestones and had a moderate ID with the severely delayed language of a few isolated words at 4.5 years. A mild bilateral sensorineural hearing loss was diagnosed, for which he has a hearing aid. He has no clinical seizure but puffs of slow theta waves in the temporal and anterior regions. He recently developed abnormal non-epileptic choreiform movements. Brain MRI revealed a slightly dysmorphic appearance of the corpus callosum at the junction of the middle and posterior thirds and a specific minimal punctiform signal anomaly in the bifrontal subcortical white matter. The de novo c.1234_1238del p.(N412Efs*7) variant in DOP1A was identified by whole-exome sequencing. The variant was not detected in either parent by Sanger sequencing.
